# Development of 2-in-1 Sensors for the Safety Assessment of Lithium-Ion Batteries via Early Detection of Vapors Produced by Electrolyte Solvents

**DOI:** 10.1021/acsami.3c03564

**Published:** 2023-05-26

**Authors:** Oleg Lupan, Nicolae Magariu, David Santos-Carballal, Nicolai Ababii, Jakob Offermann, Pia Pooker, Sandra Hansen, Leonard Siebert, Nora H. de Leeuw, Rainer Adelung

**Affiliations:** † Department for Materials Science—Functional Nanomaterials, Faculty of Engineering, Christian-Albrechts-University of Kiel, Kaiserstraße 2, D-24143 Kiel, Germany; ‡ Department of Microelectronics and Biomedical Engineering, Center for Nanotechnology and Nanosensors, Technical University of Moldova, 168 Stefan cel Mare Avenue, MD-2004 Chişinău, Republic of Moldova; § School of Chemistry, University of Leeds, Leeds LS2 9JT, United Kingdom; ∥ Department of Earth Sciences, Utrecht University, Budapestlaan 4, 3584 CD Utrecht, The Netherlands

**Keywords:** CuO, TiO_2_, heterostructures, gas sensor, battery safety, 2-in-1 sensors

## Abstract

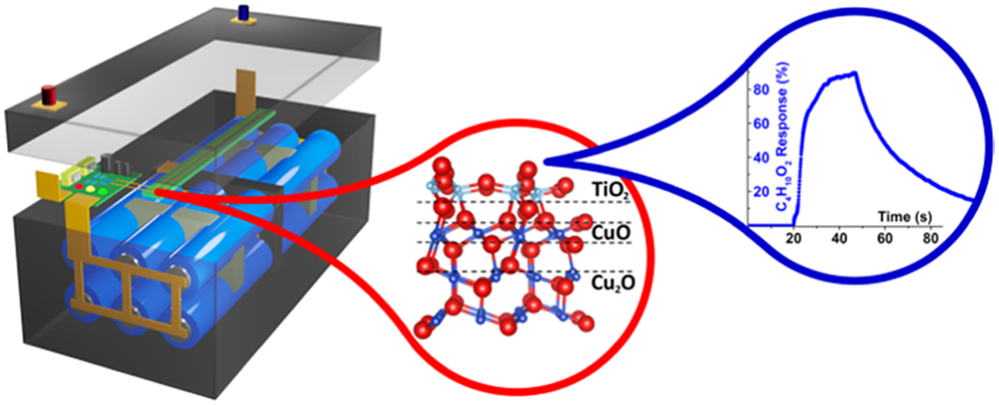

Batteries play a critical role in achieving zero-emission goals and in the transition toward a more circular economy. Ensuring battery safety is a top priority for manufacturers and consumers alike, and hence is an active topic of research. Metal-oxide nanostructures have unique properties that make them highly promising for gas sensing in battery safety applications. In this study, we investigate the gas-sensing capabilities of semiconducting metal oxides for detecting vapors produced by common battery components, such as solvents, salts, or their degassing products. Our main objective is to develop sensors capable of early detection of common vapors produced by malfunctioning batteries to prevent explosions and further safety hazards. Typical electrolyte components and degassing products for the Li-ion, Li–S, or solid-state batteries that were investigated in this study include 1,3-dioxololane (C_3_H_6_O_2_—DOL), 1,2-dimethoxyethane (C_4_H_10_O_2_—DME), ethylene carbonate (C_3_H_4_O_3_—EC), dimethyl carbonate (C_4_H_10_O_2_—DMC), lithium bis(trifluoromethanesulfonyl)imide (LiTFSI), lithium nitrate (LiNO_3_) salts in a mixture of DOL and DME, lithium hexafluorophosphate (LiPF_6_), nitrogen dioxide (NO_2_), and phosphorous pentafluoride (PF_5_). Our sensing platform was based on ternary and binary heterostructures consisting of TiO_2_(111)/CuO(1̅11)/Cu_2_O(111) and CuO(1̅11)/Cu_2_O(111), respectively, with various CuO layer thicknesses (10, 30, and 50 nm). We have analyzed these structures using scanning electron microscopy (SEM), energy-dispersive X-ray spectroscopy (EDX), micro-Raman spectroscopy, and ultraviolet–visible (UV–vis) spectroscopy. We found that the sensors reliably detected DME C_4_H_10_O_2_ vapors up to a concentration of 1000 ppm with a gas response of 136%, and concentrations as low as 1, 5, and 10 ppm with response values of approximately 7, 23, and 30%, respectively. Our devices can serve as 2-in-1 sensors, functioning as a temperature sensor at low operating temperatures and as a gas sensor at temperatures above 200 °C. Density functional theory calculations were also employed to study the adsorption of the vapors produced by battery solvents or their degassing products, as well as water, to investigate the impact of humidity. PF_5_ and C_4_H_10_O_2_ showed the most exothermic molecular interactions, which are consistent with our gas response investigations. Our results indicate that humidity does not impact the performance of the sensors, which is crucial for the early detection of thermal runaway under harsh conditions in Li-ion batteries. We show that our semiconducting metal-oxide sensors can detect the vapors produced by battery solvents and degassing products with high accuracy and can serve as high-performance battery safety sensors to prevent explosions in malfunctioning Li-ion batteries. Despite the fact that the sensors work independently of the type of battery, the work presented here is of particular interest for the monitoring of solid-state batteries, since DOL is a solvent typically used in this type of batteries.

## 1 Introduction

Batteries have become a key technology in the transition toward climate neutrality and are increasingly used in all aspects of daily life as their energy storage capacity has increased from 90 to 250 Wh kg^–1^.^1^ Batteries serve as a fundamental component of electronic devices, battery-electric or hybrid vehicles, and are likely to find even more applications in the future in industry and healthcare applications.^2,3^ Batteries play a vital role in underpinning sustainable transportation, thereby contributing toward the international goal of zero emission. However, due to this widespread use and their high energy density, battery safety is a major concern.

### 1.1 Global Demand for Next-Generation Batteries

The depletion of oil and natural gas due to their continued use in various industrial, automotive, and other sectors has made it necessary to explore alternative energy resources. Efficient energy storage, coupled with renewable energy sources, is considered important for the transition away from fossil fuels in the near future.^1^ In recent years, the number of electric cars sold worldwide has increased significantly, with over 5.1 million electric cars sold in 2018 alone.^4^ The global market for electric vehicle batteries (EVBs) was estimated to be worth US$26.5 billion in 2022 and is projected to grow to US$84.5 billion by 2030.^5^ According to an analysis by Castelvecchi,^6^ by 2035, over half of new passenger vehicles sold globally will be electric, mostly powered by EVBs, which requires new safety measures.

### 1.2 Safety Concerns and Sensing Materials

Semiconductor metal oxides are currently being intensively researched as gas sensors, owing to their excellent properties for detecting gases, vapors, or ultraviolet radiation, in a variety of relevant fields.^7,8^ Copper oxide sensors functionalized with noble metals Ag, Au, Pt, and Pd have been shown to be selective for hydrogen, ethanol, or other volatile compounds.^9−11^ Studies in the literature have reported that changing the thickness of the oxide can improve the response to ethanol^9^ and that sensors functionalized with Pd nanodots can detect low hydrogen concentrations, while sensors functionalized with Ag or Ag–Pt nanodots are selective to *n*-butanol vapors.^11^ Nanowires based on the ZnO metal oxide can also detect small hydrogen concentrations.^12^ Several research teams have also developed ethanol sensors^13−15^ based on SnO_2_ nanostructures obtained by various methods, which are selective to low concentrations of ethanol vapor, whereas not only ZnO but also titanium dioxide, tin oxide, tungsten oxide, and iron oxide have been found to be suitable for the detection of hydrogen.^16−22^ A possible route for tailoring the properties of the ordered porous Au/TiO_2_ thin films for surface-enhanced Raman scattering (SERS) sensor applications was proposed recently.^23^ Au/TiO_2_ nanostructures are quite promising in such applications since they rely on localized surface plasmon resonances (LSPRs) and SERS that are important for sensors, photocatalysis, and electrodes for batteries.^23−25^

Copper oxide deposited over zinc oxide doped with Fe impurities has also been shown to be highly selective to ethanol vapors^26^ and deposition of a polymer layer has been used to obtain a hydrogen sensor that is stable at high relative humidity. CuO/Cu_2_O/ZnO:Fe^26^ and Al_2_O_3_/ZnO^22^ sensors were also capable of detecting the vapors of lithium nitrate (LiNO_3_), 1,3-dioxolane (C_3_H_6_O_2_), and 1,2-dimethoxyethane (C_4_H_10_O_2_) components of the battery electrolyte. These multi-nanolayered sensors allow for the detection and monitoring of ecological fuels, such as ethanol and hydrogen, and electrolyte vapors from batteries, which is essential to increase safety in various applications.^20,22,26^

**Figure 1:**
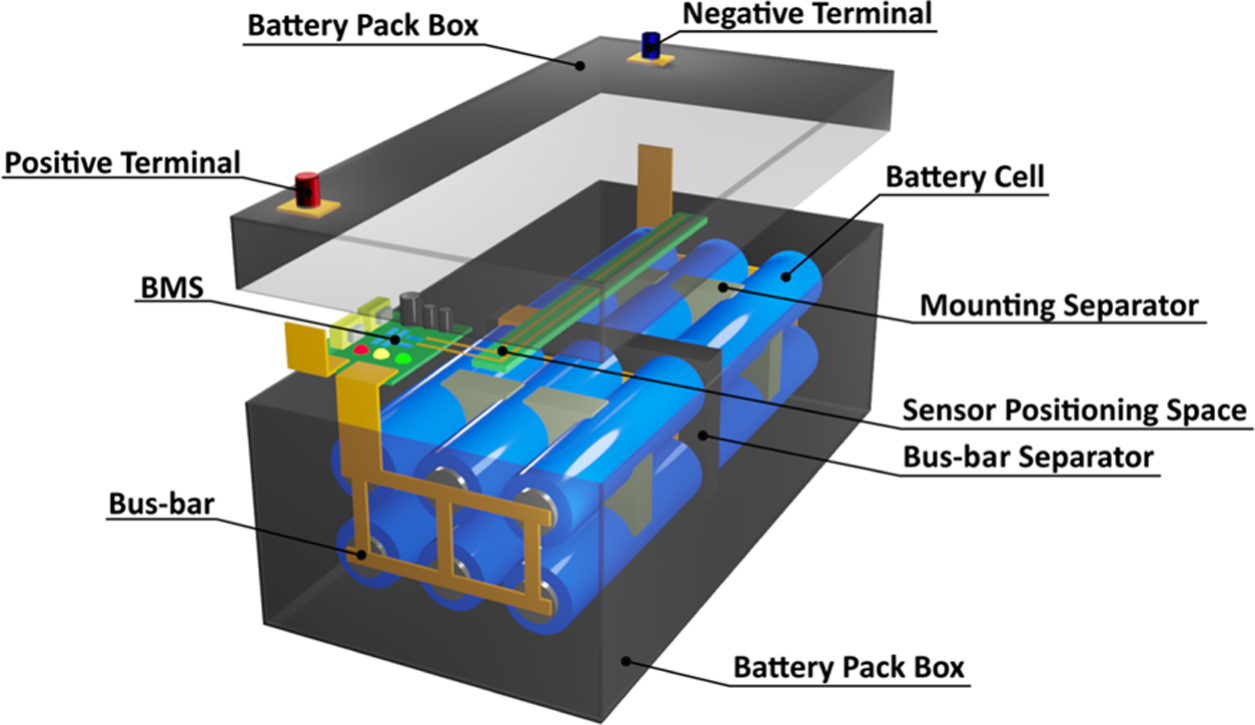
Schematic concept of the battery pack.

Battery thermal runaway (BTR), for example, is a critical process that causes rapid heat development and can produce vapors or gases.^27−30^ The vapors released during chemical decomposition in BTR will cause expansion of the batteries.^27−30^ The chemical reactions become intense at high temperatures, leading to the release of more heat and the volatilization of more components of the electrolyte, which can ultimately cause explosion of the battery unless a safety mechanism is triggered,^27,31^ at which point the erupted gases/vapor will mix with air and eventually ignite.^27−31^ The application of a gas sensor can be accomplished in different ways inside a battery in order to detect possible electrolyte leaks or decomposition products at an early stage. The simplest implementation is the installation of a gas sensor for the entire battery pack. The information from the sensor can be forwarded to the battery management system (BMS), which can interrupt the entire circuit if necessary to avoid damage and prevent the destruction of the entire battery. The schematic concepts of the battery pack are presented in Figures 1 and S1. However, this design has the limitation of not monitoring each individual cell, but the entire battery pack, which could lead to information delay between the damaged cell and the sensor. On the other hand, the installation of a sensor in each individual battery cell allows an extremely precise and short-range analysis of the battery’s condition. In addition, the health of the entire battery system can also be monitored with this set up, as the thermal runaway starts in individual battery components and spreads further within the battery cell. Thus, positioning the sensor in Li–S batteries, for example, makes sense especially near the S-cathode (see Figure S1).^28−30^ The disadvantage of having multiple sensors with the battery pack is the significantly greater technical effort required to manufacture and operate the system, as well as the further processing of information. An optimal design can involve having several gas sensors distributed and monitoring a stack of multiple battery cells.

### 1.3 Sensing in Li-Ion and Li–S Batteries

Sensors are very important for batteries in order to measure any temperature or compositional changes during operation prior to explosion.^20,22^ This study demonstrates how sensors could operate independent of the type of battery cell, showing the possibility to use them in Li-ion, Li–S as well as completely solid-state batteries.

Whereas both Li-ion and Li–S batteries contain various compounds in their electrolytes, such as solvents and various salts like DOL (1,3-dioxololane—C_3_H_6_O_2_), DMC (dimethyl carbonate—C_3_H_6_O_3_) or DME (1,2-dimethoxyethane—C_4_H_10_O_2_), and LiNO_3_, LITFSI, or LiPF_6_.^22,26^ Solid-state batteries, on the other hand, contain solid components, which are nevertheless based on the same stoichiometric components like solidified DME in combination with other salts like Li_7_P_3_S_11_.^32^ Liquid Li-ion or Li–S batteries, which have enhanced energy densities, are considered one of the solutions that can help to increase the efficient use of renewable energy resources. Electrodes with high reversible capacities have been developed, including cathode materials that can operate at high voltages and anode materials that can operate at low voltages, close to 0 V vs Li/Li^+^ ions.^33,34^

During battery operation, various vapor leaks can develop, including DME, DMC, DOL, ethyl methyl carbonate (EMC—C_4_H_8_O_3_), ethylene carbonate (EC—C_3_H_4_O_3_), and lithium nitrate (LiNO_3_).^31^ LiNO_3_ is one of the most common salt additives in Li–S batteries, important for the homogeneous formation of a solid electrolyte interface (SEI) passivation layer on the anode of the battery. LiNO_3_ also plays an important role in the development of Li–S batteries because it improves the redox reactions and the self-discharge rate. LiNO_3_ is one of the most common salt additives in Li–S batteries, important for the homogeneous formation of a solid electrolyte interface (SEI) passivation layer on the anode of the battery. Additionally, in lithium metal anodes, water reacts with lithium metal to generate H_2_ gas, hydroxides, and oxides, which have no ionic conductivity, thereby impacting the performance of the battery.^35−37^

One of the compounds that play a crucial role in all battery types named above is 1,3-dioxolane.^38^ The compound is used for polymerization reactions within the battery and on the Li anode surface to protect the anode surface from parasitic side reactions promoting the formation of a SEI layer.^38−40^ Both LiNO_3_ and DOL participate in the formation of the solid electrolyte interface.^39^ Recently, it has been shown that DOL present outside the SEI layer production is an essential compound for Li-ion and Li–S batteries. Different concentrations of DOL in combination with other compounds can be used to produce the electrolytes required by electric batteries.^40^

1,2-Dimethoxyethane in combination with Li_7_P_3_S_11_, which are solvents in Li-ion and Li–S batteries, play an important role in the mass production of solid-state batteries.^32^ A mixture of DME, DOL, and 1 M LiTFSI has been reported as one of the most suitable and widely used electrolytes for Li–S batteries.^41,42^

However, this mixture can lead to the formation of soluble polysulfides, which are responsible for undesirable reactions, such as the polysulfide shuttle.^43^ Improving the electrolyte formed by DME and DOL will increase the chemical stability and its ionic conductivity.^43^

For Li-ion batteries, LiPF_6_ is the most stable salt used in commercial electrolytes (not compatible with Li–S batteries due to a nucleophilic reaction). The salt produces PF_5_ gas during battery thermal runaway. At room temperature, LiPF_6_ can dissociate according to the following chemical reaction^44^
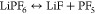
1

PF_5_ can further react with moisture leading to the formation of reactive species, such as POF_3_, which can destroy the interfacial layers on the electrolytes, dissolve some transition metals from the cathode, and ultimately decompose the electrolyte.^45,46^ To detect these battery products, different sensor structures have been proposed, but heterostructured materials are of particular interest owing to the possibility to develop portable devices for such applications.

Early detection of vapors produced by the solvents of Li-ion batteries or their degassing products, such as 1 DOL (C_3_H_6_O_2_), DME (C_4_H_10_O_2_), LiTFSI, and LiNO_3_ salts dissolved in a mixture of DOL/DME, LiPF_6_ salts, nitrogen dioxide (NO_2_), and phosphorous pentafluoride (PF_5_) released during thermal evaporation requires sensors that can send a warning to the battery management system. This allows the disconnection of the battery from the system and prevents its destruction or explosion. The pristine and noble-metal nanoparticle-functionalized CuO/Cu_2_O and TiO_2_/CuO/Cu_2_O heterojunctions have previously been used to successfully simulate the sensing properties of multi-nanolayers.^9,11^

Our study reports the reliable detection of C_4_H_10_O_2_ vapors compared to other gases such as C_3_H_6_O_2_, or degradation products of the used salts (LiNO_3_ and LiPF_6_), LiNO_3_ and LiPF_6_ described as E1 and LP30, respectively, using TiO_2_(111)/CuO(1̅11)/Cu_2_O(111) and CuO(1̅11)/Cu_2_O(111) heterojunctions, whose structures were investigated using SEM, EDX, micro-Raman, and UV–vis spectroscopy.

In this study, we analyzed heterostructures of nanolayered metal oxides and calculated the adsorption of various molecules on the exposed surface of CuO(1̅11)/Cu_2_O(111) and TiO_2_(111)/CuO(1̅11)/Cu_2_O(111) heterostructures. We have used calculations based on the density functional theory (DFT) with on-site Coulombic interactions and long-range dispersion corrections [DFT+U-D3-(BJ)]. The molecules we considered for adsorption included DOL, DME, nitrogen dioxide (NO_2_), phosphorous pentafluoride (PF_5_), and water (H_2_O). These molecules are either components or degassing products of battery electrolytes, and we also assessed the impact of humidity during operation of the gas sensor. In addition, the effects of humidity during operation of the gas sensor were evaluated. We discuss the interaction geometries and energies of the molecular species at the heterojunctions as well as charge transfers and local density of states to explain the sensitivity and selectivity of the multi-nanolayered sensors. The calculated adsorption energies, structures, and electron properties support and complement our experimental observations.

## 2 Experimental and Computational Details

CuO/Cu_2_O and TiO_2_/CuO/Cu_2_O heterostructures were synthesized on either a glass substrate from ThermoScientific (2.5 × 7.5 cm^2^) or quartz substrate (to remove the Na signal from XPS measurements, which originates due to diffusion from glass substrates),^11^ which had been cleaned using an 11% HCl solution, rinsed in distilled water and acetone, and subjected to an ultrasonic bath in ethanol.^11^ The substrate was then rinsed with deionized water, following the procedure described in previous works.^9,47^ The CuO/Cu_2_O layers with thicknesses of 10, 30, and 50 nm, which were labeled as Cu10, Cu30, and Cu50, were deposited by vacuum sputtering of metallic copper using a customized RF-magnetron system, as detailed in earlier works.^9,11^ The metal layers obtained were subjected to thermal treatment in a furnace at a temperature of 420 °C for 30 min in air, resulting in the mixed phase CuO(1̅11)/Cu_2_O(111).^10,48^ To synthesize the three-layered TiO_2_/CuO/Cu_2_O heterostructures, the ALD/sputtering/annealing approach was used to deposit the TiO_2_ layer on top of the bilayered CuO/Cu_2_O heterostructures grown according to the process described in our previous works.^9,11,49^ The three-layered TiO_2_/CuO/Cu_2_O heterostructures were subjected to thermal treatment in a furnace at 600 °C for 3 h. After two types of heterostructures were developed, Au electrodes separated by 1 mm were deposited on their surfaces using a meander-shaped mask in a vacuum atmosphere, as detailed in previous works.^9−11,47,48^ The morphology of the heterojunctions was investigated using scanning electron microscopy (SEM), generated using a REM-ZEISS device at a voltage of 7 kV and a current of 10 μA, whereas the chemical composition was measured using energy-dispersive X-ray (EDX). The EDX spectra were recorded at an accelerating voltage of 15 kV. Micro-Raman measurements were performed using a WITec Alpha300 RA Raman spectrometer to investigate the surfaces.^20^ Micro-Raman characteristics were investigated in backscattering geometry using a frequency-doubled neodymium-doped yttrium aluminum garnet (Nd:YAG) laser (10 mW) with an excitation wavelength of 532 nm.^49^ A spectrophotometer was used to characterize the optical properties of the heterojunctions. The sensor properties were investigated using a computer-controlled Keithley2400 source-meter and later processed through the LabView software (from National Instruments).

Binary CuO(1̅11)/Cu_2_O(111) and ternary TiO_2_(111)/CuO(1̅11)/Cu_2_O(111) heterostructures and their reactivity toward C_3_H_6_O_2_, C_4_H_10_O_2_, NO_2_, PF_5_, and H_2_O were simulated using spin-polarized DFT calculations, to account for open-shell systems, as implemented in the Vienna Ab Initio Simulation Package (VASP)^50−53^ with the Perdew, Burke, and Ernzerhof (PBE)^54,55^ exchange-correlation functional and a kinetic energy cut-off of 400 eV for the periodic plane-wave basis set. Geometry optimizations were carried out using a force-based Newton line optimizer, which is an efficient conjugate gradients technique^56,57^ and were stopped when the Hellmann–Feynman forces on all atoms reached the minimum threshold of 0.01 eV Å^–1^. We are confident that our structures were properly optimized, since the forces on all atoms were minimized. Full details of the calculation procedures are provided in Text S1.

The atomic charges were allocated using a fast and robust algorithm for the Bader decomposition of the electron density.^58−60^ All structural representations and charge density flows were created using the Visualization for Electronic and Structural Analysis (VESTA) program.^61^ The extraction and analysis of the projected density of states (PDOS) and charge density differences were carried out using VASPKIT.^62^ The PDOS were plotted using OriginPro.^63^

The charge density differences (Δρ) were computed as
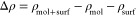
2where ρ_mol+surf_ is the charge density of the heterostructure with the interacting molecule and ρ_mol_ as well as ρ_surf_ are the charge density of the molecular and surface fragments with the same geometry of the adsorption configuration.

The adsorption energy (*E*_ads_) was calculated using the equation
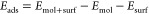
3where *E*_mol+surf_, *E*_mol_, and *E*_surf_ refer to the energy of the surface slab with the adsorbate, the energy of the isolated molecule, and the energy of the pristine interface, respectively, in their ground-state configurations. Note that the small zero-point energy (ZPE) contribution to the adsorption energy *E*_ads_ was not considered in this work. Full computational details are reported in Text S1.

**Figure 2:**
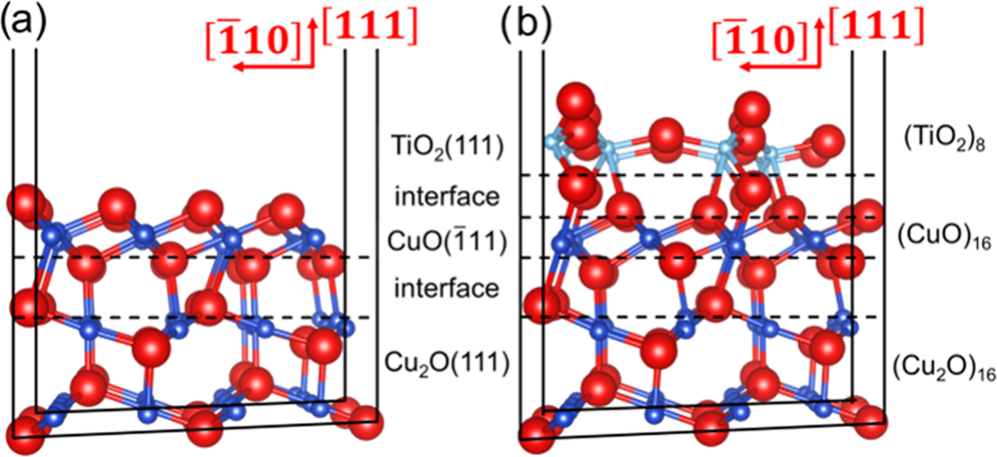
Side views of the optimized structures for (a) binary CuO(1̅11)/Cu_2_O(111) and (b) ternary TiO_2_(111)/CuO(1̅11)/Cu_2_O(111) interfaces. Crystallographic directions and stacking sequence of the atomic layers are indicated. O atoms are shown in red, Cu atoms are shown in dark blue, and Ti atoms are shown in light blue.

The binary CuO(1̅11)/Cu_2_O(111) heterostructure was simulated using a slab containing 10 formula units (f.u.) of Cu_2_O and 16 f.u. of CuO with an area of 125.512 Å^2^.^2^ We added a thin layer of 8 f.u. of TiO_2_ on the topmost surface of the binary sensor to construct the ternary TiO_2_(111)/CuO(1̅11)/Cu_2_O(111) heterojunction,^11^ as shown in Figure 2. The heterojunctions were created using the most stable terminations of the isolated Cu_2_O(111), CuO(1̅11), and TiO_2_(111) surfaces, whose slabs were symmetric, stoichiometric and nonpolar,^9^ following the Tasker approach.^64^ Similar terminations *A* of Cu_2_O(111) and CuO(1̅11) surfaces are Tasker type 2 with (O)–(Cu_4_)–(O) as the stacking for the atomic layers, whereas the only possible termination of the TiO_2_(111) surface is also Tasker type 2, with the closely packed (O)–(Ti)–(O) layers forming almost single planes. Despite the different thicknesses of the computational model and experimental samples, we are confident that our calculations correctly capture the underlying chemistry of the systems under study and allow us to draw meaningful conclusions.^9,11,22,65−67^ A vacuum gap of 20 Å was added in the direction perpendicular to the surface to avoid the interaction between neighboring cells. The lower portion of the calculation cells consisted of two stacking sequences that were maintained in their optimized bulk positions to mimic the bulk phases, whereas the remaining layers were allowed to relax.

The tested battery solvents are C_3_H_6_O_2_ (DOL) and C_4_H_10_O_2_ (DME). Furthermore, two battery electrolyte mixtures were analyzed, i.e., a mixture of DME and DOL (50:50 (v/v)) with 1 M LiTFSI and 0.25 M LiNO_3_, which we will call “E1”. In addition, a standard electrolyte “LP30” is tested, containing equally ethylene carbonate and dimethyl carbonate (50:50 (v/v)) as well as 1 M PF_6_. All chemicals were obtained from Sigma-Aldrich.

## 3 Results and Discussion

### 3.1 Morphological Analyses

**Figure 3:**
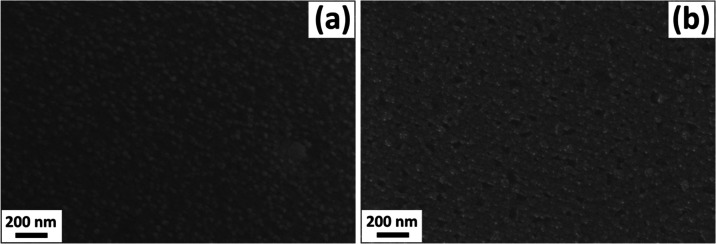
SEM images of (a) nanocrystallite CuO/Cu_2_O samples and (b) TiO_2_/CuO heterostructures with the CuO layer thickness of 10 nm (Cu10).

Figure 3a presents the SEM micrograph of the nanocrystalline CuO/Cu_2_O specimens with a thickness of 10 nm (sample set labeled as Cu10) grown on a glass substrate using a reproducible ALD/sputtering/annealing approach,^9^ followed by thermal annealing at 420 °C for 30 min in air. The layered thin films adhere strongly to the microscopic glass substrates and appear with a morphology of films composed of nanocrystallites. Figure 3b presents the SEM images of the TiO_2_/CuO heterostructures, which reveal that the nanocrystallites shown in Figure 3a are coated with a layer of TiO_2_, consisting of nanogranules/dots, as confirmed in the elemental map shown in the analysis section in Figures S2 and S3. The schematic sectional view of the ternary TiO_2_(111)/CuO(1̅11)/Cu_2_O(111) heterojunction is presented in Figure S4.

### 3.2 Micro-Raman Characterization

**Figure 4:**
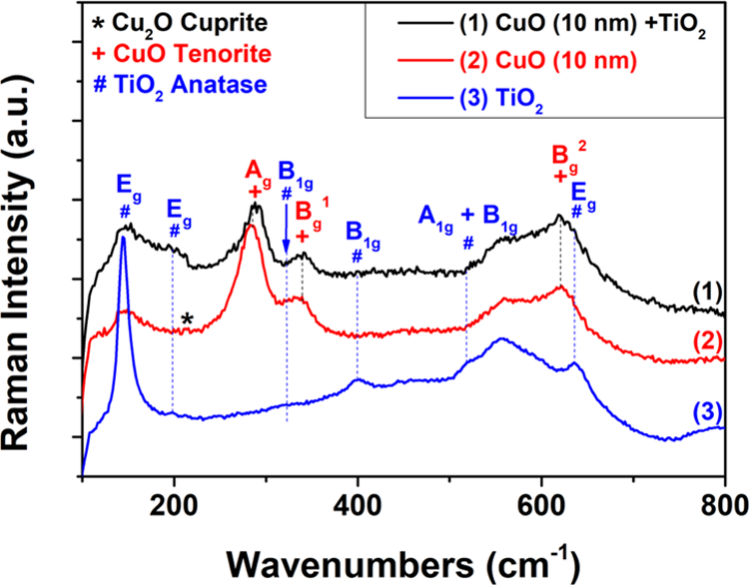
Micro-Raman spectra of thin nanocrystalline layers of (1) TiO_2_/CuO heterostructures (Cu10), (2) CuO/Cu_2_O films (Cu10), and (3) TiO_2_ layer.

Micro-Raman spectroscopy was conducted with a WITec system to determine the chemical composition of the sensor materials and to study the lattice dynamics (electron–phonon interaction) of the CuO/Cu_2_O and TiO_2_/CuO nanomaterials at the nanoscale. The micro-Raman spectra were obtained at room temperature in the range 90–900 cm^–1^ for TiO_2_/CuO, CuO/Cu_2_O, and TiO_2_ nanomaterials, as shown in Figure 4. The copper oxide films (Cu10) (curve 2) exhibit both the CuO tenorite phase and the Cu_2_O cuprite phase (marked with an asterisk “*”), but in the TiO_2_/CuO heterostructures (Cu10) (curve 1), the cuprite phase vanishes since the CuO/Cu_2_O layer has a thickness of only 10 nm (Cu10). After deposition of the TiO_2_ layer, the heterostructures undergo another annealing process that transforms the cuprite phase into the tenorite phase. CuO was detected in the highly sensitive micro-Raman characterization of the annealed samples. For the TiO_2_/CuO samples, we found modes at about 282, 333, and 611 cm^–1^, which correspond to CuO and additionally at 130, 215, and 628 cm^–1^, which correspond to Cu_2_O in the CuO/Cu_2_O sample set. Tenorite, which has 12 phonon branches due to its four atoms in the primitive cell, has the following zone-center modes^68−70^
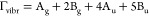
4where A_g_ + 2B_g_ – 9 are the optical modes, which are Raman active, 3A_u_ + 3B_u_ are the six IR-active modes, and A_u_ + 2B_u_ are the three acoustic modes.^68−70^ The IR modes involve the vibration of Cu and O atoms, which induces a dipole moment along the *b*-axis for the A_u_ modes and perpendicular to this axis for the B_u_ modes.^68−70^ Cu_2_O is symmetrical with the space group *Pn*3̅*m* and its unit cell has two formula units (Cu_4_O_2_).^68−70^ Cuprite, which is very symmetrical, has the space group *Pn*3̅*m* and its unit cell contains two formula units (Cu_4_O_2_).^68−70^ Cuprite, which possesses six atoms in the unit cell, has 18 modes at the Γ point^68−70^
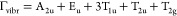
5

where T_1u_ modes are infrared active and are associated with the vibration of the copper and oxygen lattices and consist of the Cu–O stretching mode and asymmetric O–Cu–O bending mode.^71^ The anatase phase, which belongs to the *D*_4*h*_^19^ (*I*4/*amd*) space group has 2TiO_2_ formula units per primitive cell. According to the group analysis, the optical modes at the Γ point are^72^
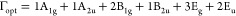
6where A_1g_, B_1g_, and E_g_ are Raman active modes and the A_2u_ mode as well as the 2E_u_ modes are infrared active. The peaks at ∼144, ∼197, ∼326, ∼400, ∼517, and ∼ 635 cm^–1^ can be attributed to E_g_, E_g_, B_1g_, B_1g_, A_1g_ + B_1g_, and E_g_ modes, respectively; see Figure 4.^72,73^

### 3.3 Ultraviolet, Visible, and Near-Infrared Spectroscopy Studies

**Figure 5:**
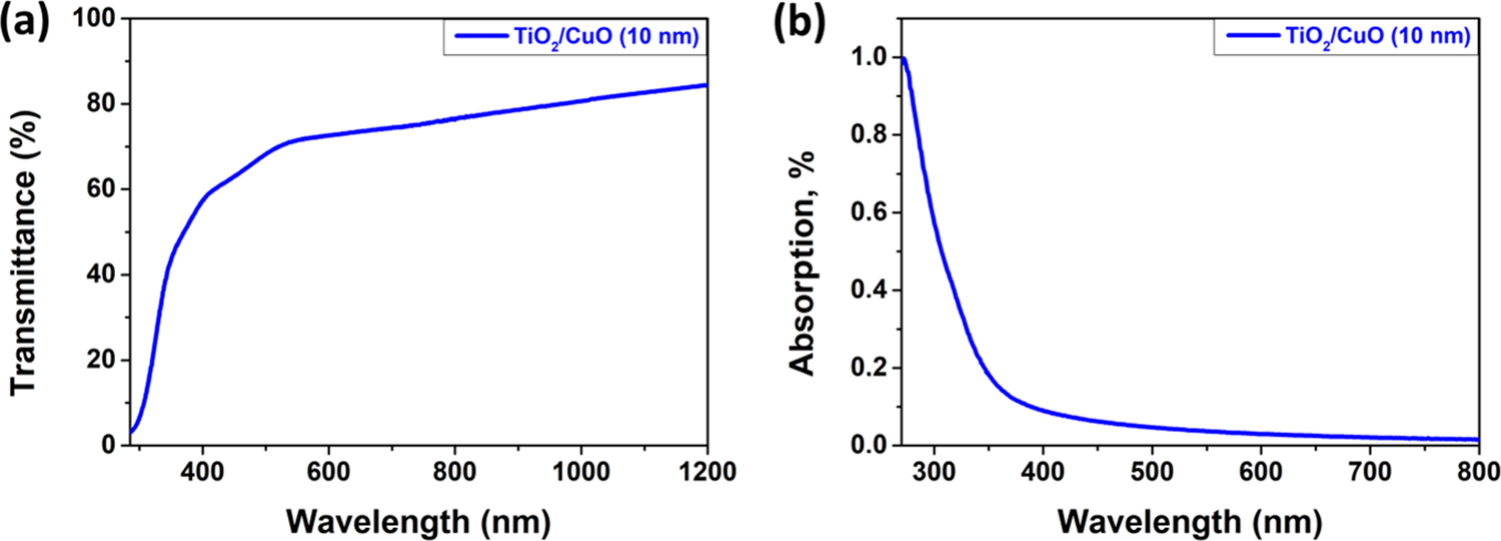
(a) Transmission spectra and (b) plot of absorption near the UV edge vs wavelength for the TiO_2_/CuO heterostructure with a thickness of the CuO layer of 10 nm (Cu10), after thermal treatment at 420 °C for 30 min.

Ultraviolet, visible, and near-infrared (UV–vis–NIR) absorption spectroscopy is an investigation technique used to determine the energy levels and optical characteristics of semiconductor or oxide nanomaterials. The transmission and absorption spectra are presented in Figure 5. Figure 5a shows the transmission spectra of the TiO_2_/CuO layered structures with a thickness of 10 nm (Cu10) annealed at 420 °C for 30 min. We found that the TiO_2_/CuO specimens possess normal transmission over 72% beyond a wavelength of 620 nm, as the main mechanism of light trapping in the thin absorbent TiO_2_ layers involves the scattering of light.^74^ The absorption vs wavelength spectrum indicates only one absorption peak centered at around 250–400 nm (Figure 5b).

**Figure 6:**
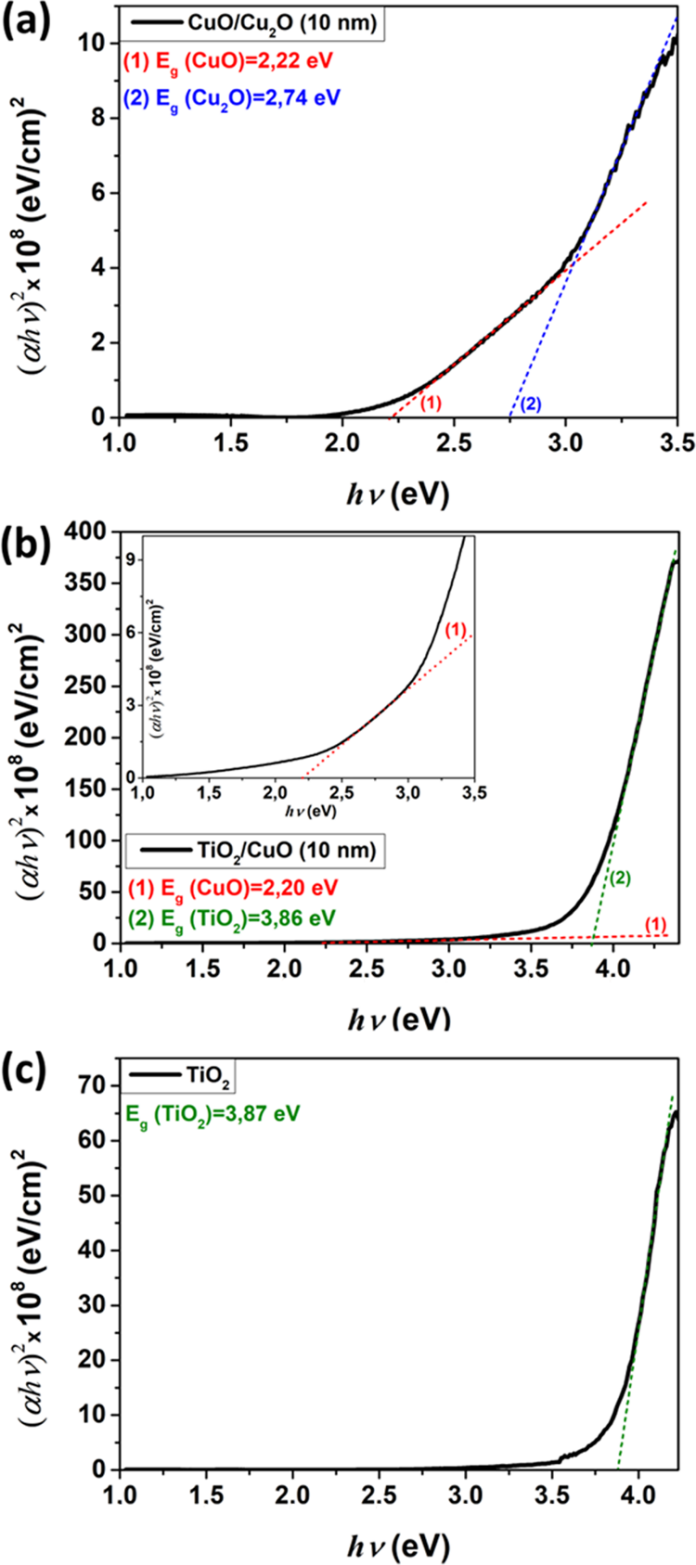
Plot of (α*h*ν)^2^ vs *h*ν for (a) CuO/Cu_2_O (Cu10); (b) TiO_2_/CuO (Cu10); and (c) TiO_2_.

The spectra were measured at room temperature for CuO/Cu_2_O (Cu10) and TiO_2_/CuO (Cu10) nanolayers to observe the optical absorption and excitonic transition characteristics of the specimens. As estimated from the plots presented in Figure 6a for CuO/Cu_2_O films, the energy gap (*E*_g_) of the CuO thin nanolayers is around 2.22 eV and for Cu_2_O it is about 2.74 eV. The best linear fit was found for a direct permitted optical transition in the copper oxide nanostructures.^75,76^ In the case of TiO_2_/CuO heterostructures (Figure 6b), the optical energy gaps *E*_g_ of CuO and TiO_2_ thin films are about 2.20 and 3.86 eV, respectively. However, the electronic band gap vanishes for Cu_2_O, which is in agreement with the Raman experiments for these heterostructures. Figure 6c presents a plot of (α*h*ν)^2^ vs *h*ν for TiO_2_, from which we deduced an *E*_g_ value of about 3.87 eV.

### 3.4 Compositional Analysis

Energy-dispersive X-ray spectroscopy (EDX) was performed at 15 kV in combination with SEM to determine the composition of the nanolayered structures.^77^

Figures S2 and S3 present the EDX elemental mapping compositional images of the CuO layer and TiO_2_/CuO heterostructures after being treated at 600 °C for 3 h. Figure S2 clearly shows a uniform distribution of O and Cu elements in a region of the CuO layer with a thickness of 10 nm (Cu10) (Figure S2b,c).

Figure S3 shows a region of the TiO_2_/CuO heterostructure with a CuO thickness of 10 nm (Cu10), revealing a uniform distribution of Ti, Cu, and O elements (Figure S3b–d). These results were further confirmed by EDX line scans taken along the TiO_2_/CuO heterostructure, which are shown in Figure S5. However, the small size of the individual Ti particles and the limited resolution of SEM/EDX prevented their accurate identification.

### 3.5 Gas-Sensing Properties

**Figure 7:**
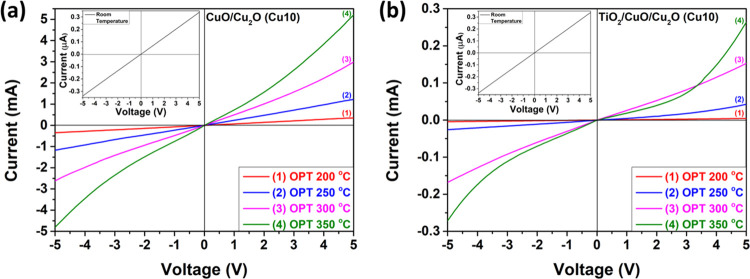
Current–voltage (*I*–*V*) characteristics of (a) CuO/Cu_2_O samples and (b) TiO_2_/CuO/Cu_2_O heterostructures with thicknesses of the CuO layer of 10 nm (Cu10), measured at different temperatures.

Figure 7a illustrates the current–voltage (*I*–*V*) characteristics of the CuO/Cu_2_O samples and Figure 7b shows the same property for the TiO_2_/CuO/Cu_2_O samples with thicknesses of 10 nm (Cu10) at different temperatures. At room temperature, both types of samples exhibit linear characteristics, but as the operating temperature increases, an Ohmic contact behavior is observed. Furthermore, the currents for the TiO_2_/CuO/Cu_2_O (Cu10) sample (Figure 7b) are lower than for the CuO/Cu_2_O (Cu10) sample (Figure 7a) at all operating temperatures due to the TiO_2_ layer, which increases the initial resistance of the sample.

**Figure 8:**
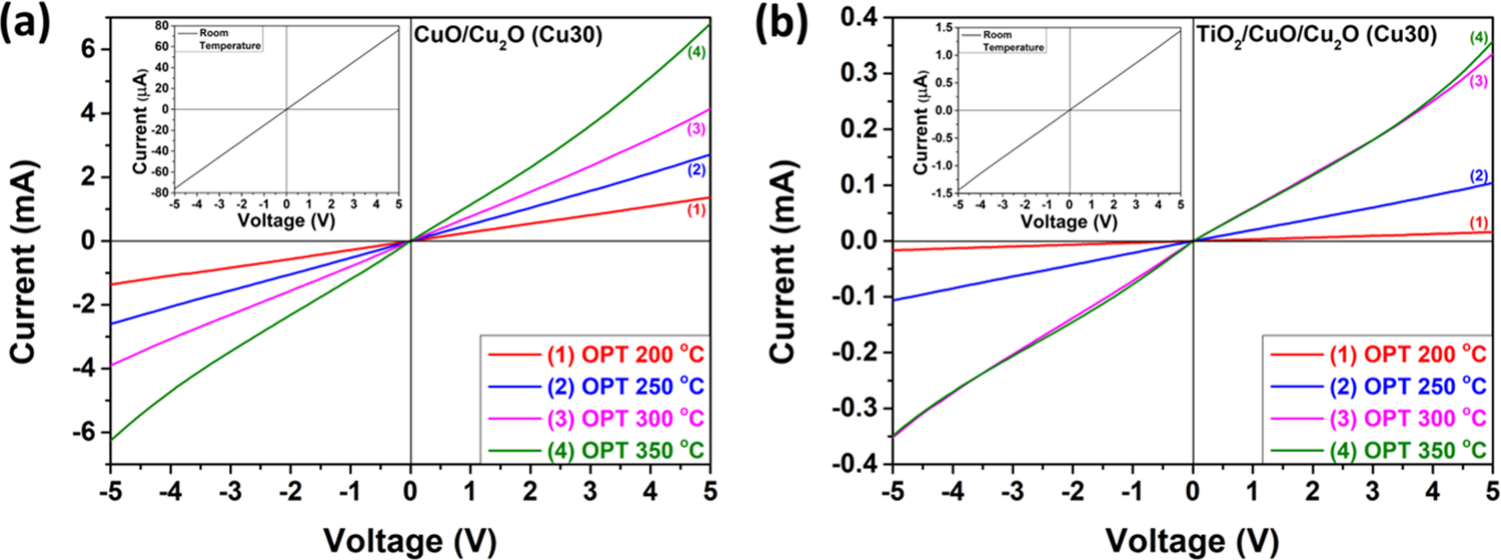
Current–voltage (*I*–*V*) characteristics of (a) CuO/Cu_2_O and (b) TiO_2_/CuO/Cu_2_O samples with thicknesses of the CuO layer of 30 nm (Cu30), measured at different temperatures.

**Figure 9:**
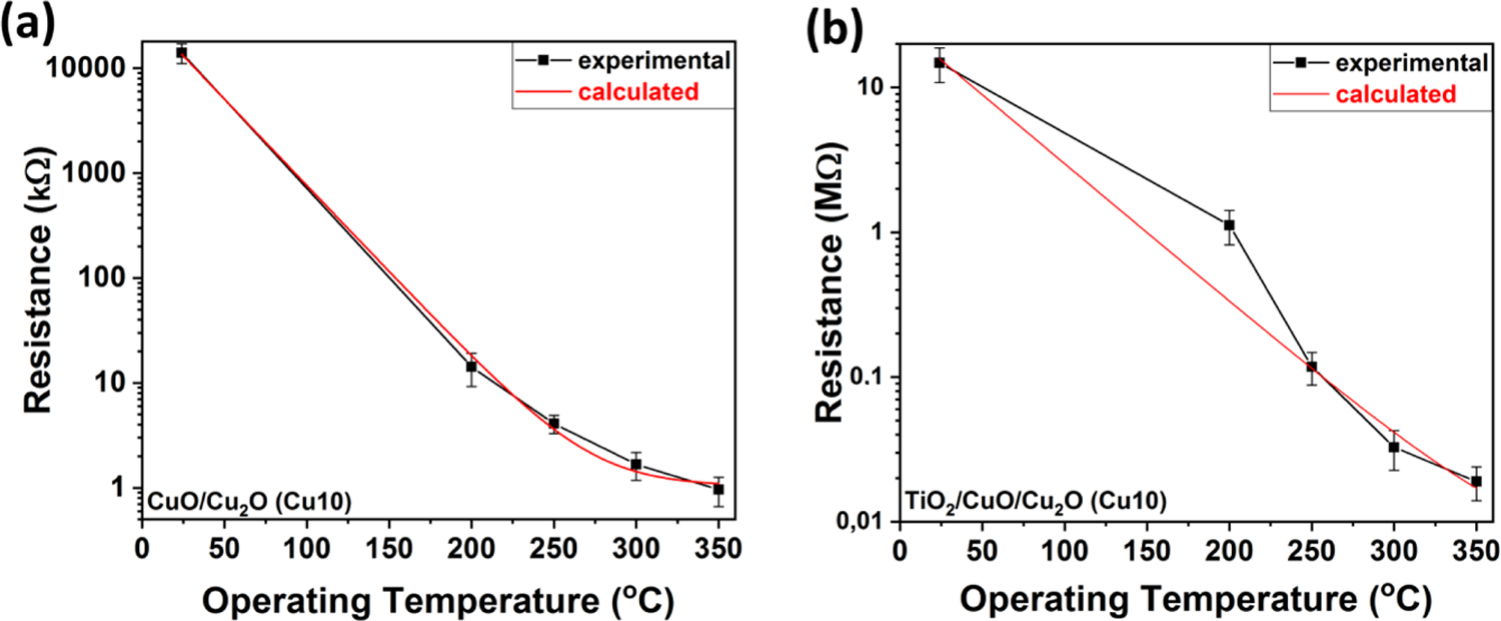
Plot of electrical resistance versus operating temperature for (a) CuO/Cu_2_O (Cu10) and (b) TiO_2_/CuO/Cu_2_O (Cu10) samples.

Figure 8a,b illustrates the *I*–*V* characteristics of CuO/Cu_2_O and TiO_2_/CuO/Cu_2_O samples with thicknesses of 30 nm (Cu30), respectively, measured at different temperatures. The plots show that both types of samples exhibit linear *I*–*V* characteristics at room temperature. The samples show Ohmic contact behavior with an increase in operating temperature. We also found that the currents for the TiO_2_/CuO/Cu_2_O (Cu30) sample (Figure 8b) are lower than for the CuO/Cu_2_O (Cu30) sample (Figure 8a) at all operating temperatures due to the TiO_2_ layer, which increases the initial resistance of the sample. The *I*–*V* characteristics of the CuO/Cu_2_O and TiO_2_/CuO/Cu_2_O layered structures with thicknesses of 50 nm (Cu50) at various temperatures are represented in Figure S6. It can be concluded that for CuO/Cu_2_O samples (Cu10, Cu30, and Cu50) at high operating temperatures (350 °C), the *I*–*V* characteristics become less Ohmic, whereas for the TiO_2_/CuO/Cu_2_O samples (Cu10, Cu30, and Cu50), this change is observed at lower operating temperatures (250 °C). The different *I*–*V* characteristics of the binary and ternary heterojunctions are due to the different electrical resistances of the samples represented in Figures 9 and S7, leading to changes in the electrical resistance and due to a change in selectivity from one gas to another depending on the sample.

Figure 9a,b shows the dependence of the electrical resistance versus the operating temperature of CuO/Cu_2_O (Cu10) (Figure 9) and TiO_2_/CuO/Cu_2_O (Cu10) samples. The investigated samples (black line) display a decrease in electrical resistance (red line), which is typical for semiconducting metal oxides.

The resistance (red line) displayed in Figure 9 was calculated as
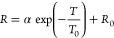
7where *R* is the calculated resistance, α is the temperature coefficient, *T* is the operating temperature, *T*_0_ is the initial temperature, and *R*_0_ is the initial resistance.

The electrical resistance of the sensors based on the CuO/Cu_2_O (Cu10) heterostructures is of the order of kΩ. After depositing the TiO_2_ layer, an increase in electrical resistance was observed, which reached the order of MΩ. However, despite increasing the Cu layer thickness to 30 nm (Cu30) and 50 nm (Cu50) and even after TiO_2_ deposition, a decrease in resistance was observed as seen in Figure S7. Thus, our heterojunctions can function as a 2-in-1 device, serving as both a temperature sensor at low operating temperatures and a gas sensor at temperatures above 200 °C.

**Figure 10:**
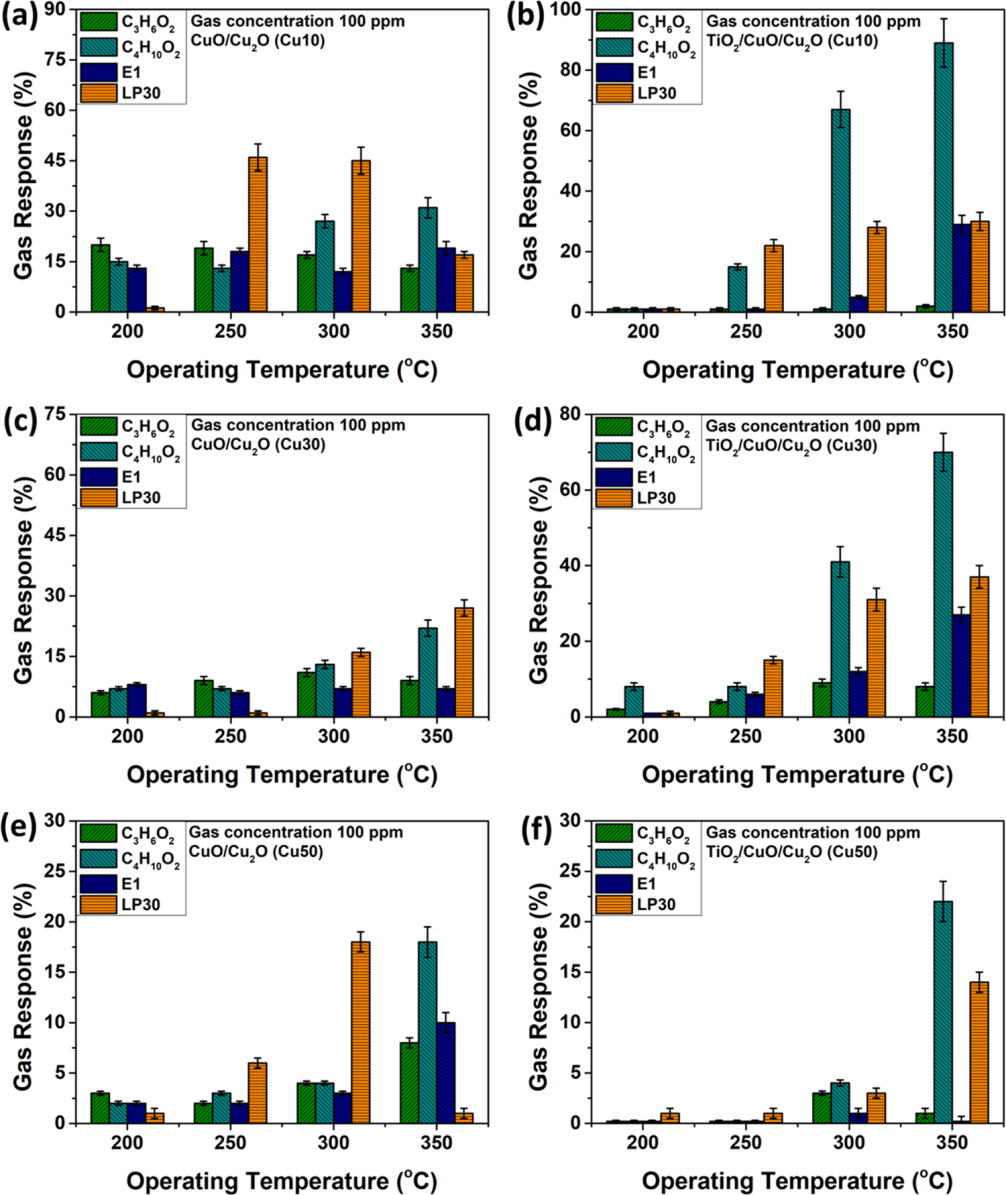
Gas response (C_3_H_6_O_2_, C_4_H_10_O_2_, E1, and LP30) vs operating temperatures for (a, c, e) CuO/Cu_2_O and (b, d, f) TiO_2_/CuO/Cu_2_O samples with different thicknesses of 10 nm (Cu10), 30 nm (Cu30), and 50 nm (Cu50).

Figure 10 depicts the response of CuO/Cu_2_O (Figure 10a,c,e) and TiO_2_/CuO/Cu_2_O samples (Figure 10b,d,f) with thicknesses of 10 nm (Cu10), 30 nm (Cu30), and 50 nm (Cu50) to 100 ppm of C_3_H_6_O_2_, C_4_H_10_O_2_, E1, and LP30 as a function of operating temperatures. Figure 10a,c,e shows that all thicknesses of the CuO/Cu_2_O samples respond to all gases, with a higher selectivity toward LP30, especially for CuO/Cu_2_O specimens with 10 nm thickness (Cu10) at OPTs 250 and 300 °C with responses of ∼46 and ∼45%, respectively. For the TiO_2_/CuO/Cu_2_O samples (Figure 10b,d,f), the selectivity changes to C_4_H_10_O_2_ with the highest responses observed at operating temperatures of 300 and 350 °C. At 300 °C, the response values are ∼67, ∼41, and ∼4% for Cu10, Cu30, and Cu50, respectively, while at 350 °C, the response values are ∼89, ∼70, and ∼22% for the same samples.

**Figure 11:**
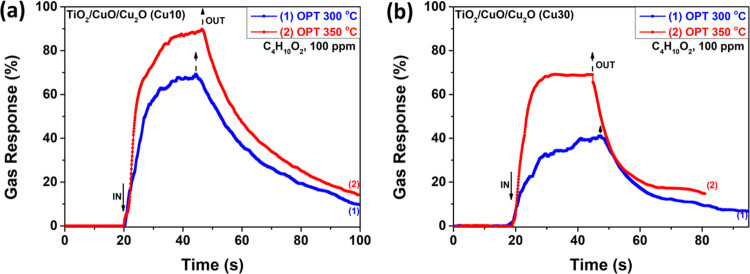
Dynamic response of TiO_2_/CuO/Cu_2_O with thickness of (a) 10 nm (Cu10) and (b) 30 nm (Cu30) at operating temperatures of 300 and 350 °C to 100 ppm of C_4_H_10_O_2_ vapors.

Figure 11 shows the dynamic response of TiO_2_/CuO/Cu_2_O samples, with thicknesses of 10 nm (Figure 11a) and 30 nm (Figure 11b), to 100 ppm of C_4_H_10_O_2_ vapors at operating temperatures of 300 and 350 °C. The highest response is observed at an operating temperature of 350 °C, with a response value of ∼89% and response/recovery times of τ_r_ ≈ 11.1 s and τ_d_ ≈ 56 s, respectively. At 300 °C, the response is ∼67% with response/recovery times of τ_r_ ≈ 11 s and τ_d_ ≈ 53.3 s, respectively. Figure 11b shows that the highest response is observed at an operating temperature of 350 °C with a response value of ∼70% and response/recovery times of τ_r_ ≈ 4 s and τ_d_ > 40 s, respectively. At 300 °C, the response is ∼41% with response/recovery times of τ_r_ ≈ 19.2 s and τ_d_ ≈ 51.1 s, respectively. Table S1 lists the types of gas sensors suitable for detecting the gases released by batteries.

**Figure 12:**
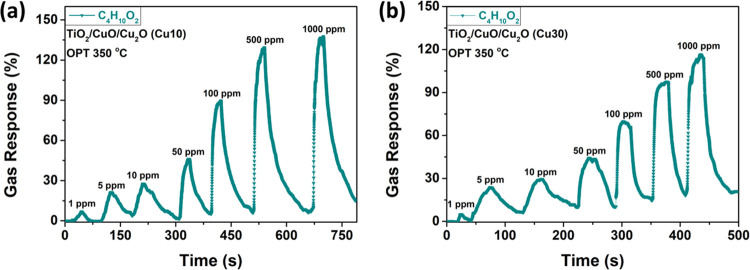
Dynamic response of TiO_2_/CuO/Cu_2_O samples with thicknesses of (a) 10 nm (Cu10) and (b) 30 nm (Cu30) at an operating temperature of 350 °C to 1, 5, 10, 50, 100, 500, and 1000 ppm of C_4_H_10_O_2_ vapors.

Figure 12 shows the dynamic response of TiO_2_/CuO/Cu_2_O samples, with thicknesses of 10 nm (Cu10) and 30 nm (Cu30), to different concentrations of C_4_H_10_O_2_ vapors (1, 5, 10, 50, 100, 500, and 1000 ppm) at an operating temperature of 350 °C. Even at low concentrations (1 ppm), the samples can detect these vapors, with a response value of ∼7%. We found that the response values increase with concentration, with the response value of 136% observed at the highest vapor concentration (100 ppm) for TiO_2_/CuO/Cu_2_O samples with 10 nm thickness (Figure 12a) and a response value of 116% for the TiO_2_/CuO/Cu_2_O samples with 30 nm thickness (Figure 12b).

### 3.6 Proposed Reactions and Sensing Mechanisms

Electric batteries use different solvents and salts as electrolytes and degassing products, such as dimethyl carbonate (DMC), ethyl methyl carbonate (EMC), diethyl carbonate (DEC), ethylene carbonate (EC), 1,2-dimethoxyethane (DME), 1,3-dioxolane (DOL), LiPF_6_, LiTFSI, and LiNO_3_ salts. These compounds can also be mixed, e.g., DOL/DME.^78^

The electrolyte in lithium cells must be anhydrous for a long life and have high conductivity and stability over a fairly wide range of temperatures and voltages.^79^

It is known that LiPF_6_ salt used in Li-ion batteries was the first to be commercialized and remains the most used salt for these batteries^80^ because of its combination of well-balanced properties, with concomitant compromises and restrictions. Two of these well-known properties of the LiPF_6_ salt are its poor stability and reactivity with water.^78,80,81^

At room temperature, solid LiPF_6_ salt dissolves in aprotic solvents to form electrolytes and it can be in equilibrium with its decomposition products, see chemical equation 1,^80,82^ whereas a schematic is presented in Figure S8.

Upon contact with H_2_O molecules, LiPF_6_ forms HF and other products following the hydrolysis process^80^
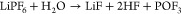
8

Since phosphoryl fluoride (POF_3_) is a reactive compound, it readily undergoes further hydrolysis according to the following equation^83^
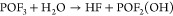
9

The result of eq 9 leads to the generation of additional HF and difluorophosphoric acid (see Figure S8), which further reacts very slowly with water^84^
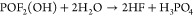
10

HF is a highly toxic and corrosive compound in the form of a gas or aqueous solution. Inhaling only a few ppm of HF owing to a battery leak, for example, in the passenger compartment of a car, can lead to acute intoxication.^83,85^ It is therefore essential to detect LiPF6 vapors instantly to prevent any negative effects.

Like salts, the electrolytes used in batteries, such as DME, also play a very important role during the operation of batteries and can lead to thermal runaway when interacting with lithium metal^79^ following the below equation
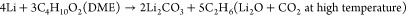
11

At high temperatures (>400 °C), the generation of oxygen that takes place inside the cell and the combustion reactions with the involvement of oxygen must also be taken into account.^79^ Thus, in the case of a venting cell, the oxygen from the outside is readily available for exothermic reactions, e.g., in the case of 1,2-dimethoxyethane (see Figure S8)^79^
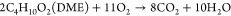
12

Great care must be taken in using high-purity salts and solvents to avoid introducing moisture during the mixing or filling processes in the electric batteries.^79^

Monitoring salts, electrolyte solvents and their degassing products strictly is crucial for correct and high-performance system management, which is necessary to prevent any threat of battery destruction and its consequences. Semiconductor metal-oxide-based sensors are promising for the early detection of battery leaks of salts, electrolyte solvents, and degassing products. In this study, we demonstrate the detection properties of CuO/Cu_2_O and TiO_2_/CuO/Cu_2_O layered structures to vapors of DOL (C_3_H_6_O_2_), DME (C_4_H_10_O_2_), LiPF_6_, and LiNO_3_ salts.

Previous studies have shown^9^ that a hole accumulation layer/zone (HAL) is formed on the surface of these metal oxides at low temperatures, and oxygen ions can be adsorbed onto the surface in different forms, e.g., as O^2–^, O^–^, and O_2_^–^ species, at temperatures between 20 and 500 °C. At high temperatures of 300–350 °C, the oxygen ions/species are in the form of O^–^ on the surface of the heterostructure. The reactions taking place on the surface affect the width of the HAL for the CuO phase between 5.2 and 16.5 nm, as shown in previous works.^10,86,87^ The largest responses were observed in CuO/Cu_2_O and TiO_2_/CuO/Cu_2_O heterostructures, with CuO thickness of 10 nm (Cu10), which is of the order of the Debye length.^10,88^ Equations for the formation of oxygen on the surface of the heterostructure are provided below
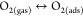
13
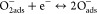
14

Applying LiPF_6_ vapors on the surface of the CuO/Cu_2_O nanostructure releases different oxygen species and forces the decomposition of the electrolyte into H_2_O, CO, and CO_2_ molecules.^89^

When C_4_H_10_O_2_ vapor is applied/adsorbed to the surface of the three-layered TiO_2_/CuO/Cu_2_O structure, an exothermic reaction takes place that results in CO_2_ and H_2_O molecules
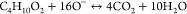
15

The negative effects caused by water in batteries have already been mentioned above.

### 3.7 Adsorption of Molecules

To explain the results of our gas response experiments, we have carried out systematic first-principles calculations to study the interaction of the surfaces of our sensors, i.e., CuO(1̅11)/Cu_2_O(111) and TiO_2_(111)/CuO(1̅11)/Cu_2_O(111) with C_3_H_6_O_2_, C_4_H_10_O_2_, NO_2_, PF_5_, and H_2_O. For the organic molecules, as well as H_2_O and PF_5_, we have investigated the adsorption by initially coordinating the negatively charged O or F with the surface Cu and Ti cations. For NO_2_, we positioned the N atom to interact directly with the O anions exposed at the surfaces. We allowed the H atoms of H_2_O to form hydrogen bonds with the surface O ions, whereas the O atoms of NO_2_ could also coordinate the under-coordinated cations of the facets. The atoms interacting at the interfaces were initially placed around 1.5 Å apart, before optimizing the energies and geometries of the entire systems, in accord with earlier studies.^65−67^

Table 1 displays the binding energies (*E*_ads_) calculated for the molecular adsorptions onto the CuO(1̅11)/Cu_2_O(111) heterojunction and three-layered TiO_2_(111)/CuO(1̅11)/Cu_2_O(111) heterostructure. The adsorption energy of −1.354 eV shows that PF_5_ interacts more strongly with the ridge 3-fold O position of the CuO(1̅11)/Cu_2_O(111) heterostructure than any of the other gases tested. The NO_2_ molecule releases only a slightly larger adsorption energy on the ridge 3-fold O site than either C_3_H_6_O_2_ or C_4_H_10_O_2_ on the 3-fold Cu atom, with the thermodynamic preference of these adsorbates toward the binary heterojunction within the range of −1.070 and −0.995 eV. In contrast, H_2_O has the lowest binding energy *E*_ads_ = −0.867 eV calculated for the 3-fold Cu site of the CuO(1̅11)/Cu_2_O(111) heterostructure. Despite the clear thermodynamic preference by at least 228 meV for all adsorbates toward one of the adsorption sites, we found a negligible difference in adsorption energies for C_4_H_10_O_2_ at the two exposed Cu atoms. The decreasing order of binding energies calculated on the ternary TiO_2_(111)/CuO(1̅11)/Cu_2_O(111) heterojunctions is
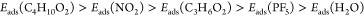
16

**Table 1:** Adsorption Energies (*E*_ads_) and Charge Transfers (Δ*q*) for C_3_H_6_O_2_, C_4_H_10_O_2_, NO_2_, PF_5_, and H_2_O on CuO(1̅11)/Cu_2_O(111) and TiO_2_(111)/CuO(1̅11)/Cu_2_O(111) Heteroepitaxial Junctions^a^

	CuO(1̅11)/Cu_2_O(111)			TiO_2_(111)/CuO(1̅11)/Cu_2_O(111)		
adsorbate	site	*E*_ads_ (eV)	Δ*q* (e)	site	*E*_ads_ (eV)	Δ*q* (e)
C_3_H_6_O_2_	3-fold Cu	–1.048	0.111	Ti	–0.919	0.102
	4-fold Cu	–0.820	0.029			
C_4_H_10_O_2_	3-fold Cu	–0.995	0.122	O	–3.707	1.223
	4-fold Cu	–0.997	0.049			
NO_2_	ridge 3-fold O	–1.070	0.087	O	–1.685	0.128
PF_5_	4-fold Cu	–0.410	–0.024	O	–0.783	–0.090
	ridge 3-fold O	–1.354	–0.142			
H_2_O	3-fold Cu	–0.867	0.079	Ti	–0.580	0.081
	4-fold Cu	–0.521	0.024			

a The adsorption site of the adsorbate is also indicated. A negative value of Δ*q* denotes that the adsorbate gains electron charge.

The most favorable adsorption configurations were calculated for C_4_H_10_O_2_ and NO_2_ on the ternary heteroepitaxial junction, whereas the largest strength of interaction for C_3_H_6_O_2_, PF_5_, and H_2_O was obtained at CuO(1̅11)/Cu_2_O(111). The trend of the binding energies observed for the adsorbates on the binary and ternary heterojunctions is consistent with our gas response experiments. Competitive adsorption of H_2_O and the other molecules considered in this study implies that humidity will not reduce the performance of our two sensor models.

**Figure 13:**
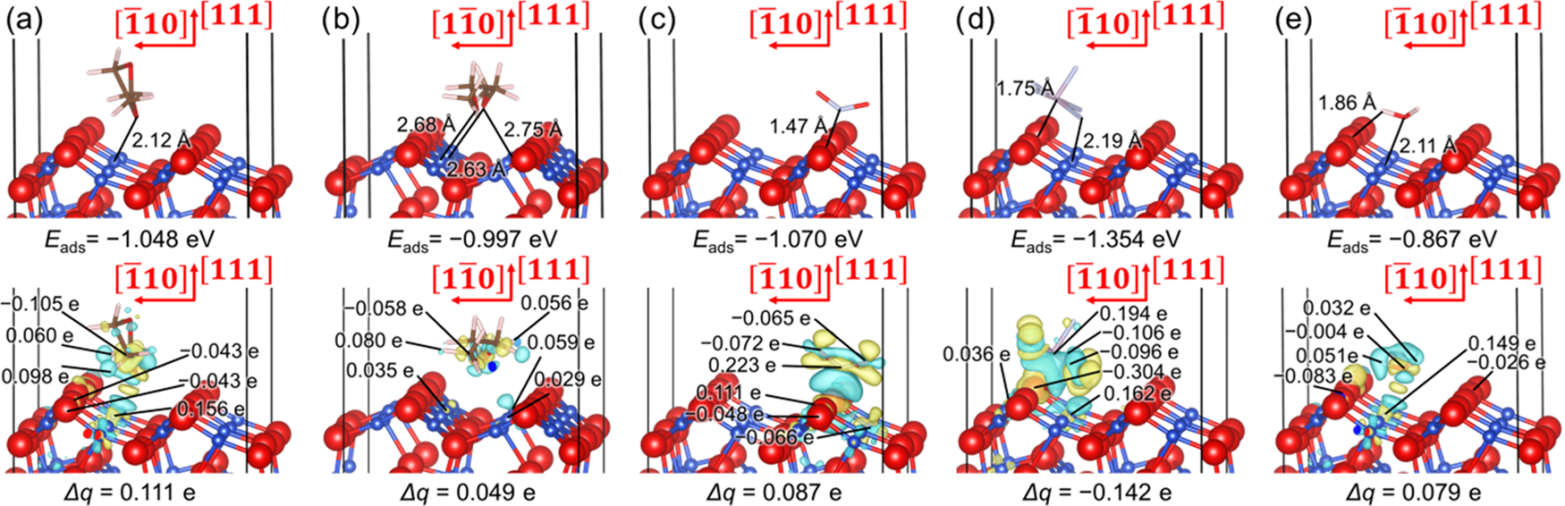
Adsorption of (a) C_3_H_6_O_2_, (b) C_4_H_10_O_2_, (c) NO_2_, (d) PF_5_, and (e) H_2_O on the CuO(1̅11)/Cu_2_O(111) heterojunction. Interatomic distances are shown in the top panels, while the charge density flow (Δρ) is represented in the bottom panels. Electron density gain and depletion regions are shown in yellow and green, respectively. Isosurfaces display a value of ±0.003 e Å^–3^. Charge transfers (Δ*q*) and atomic charge density differences for the atoms that suffered the largest change are also indicated. A negative value of charge transfer denotes that the adsorbate gains electron charge. Crystallographic directions are indicated with respect to the Cu_2_O(111) substrate. Binary heterostructures are displayed using the ball-and-stick representation, whereas the adsorbates are shown using the stick representation. O atoms are shown in red, H atoms are shown in white, C atoms are shown in brown, F atoms are shown in light blue, P atoms are shown in pink, and Cu atoms are shown in dark blue.

The configurations with the largest calculated adsorption strengths for C_3_H_6_O_2_, C_4_H_10_O_2_, NO_2_, PF_5_, and H_2_O on the binary CuO(1̅11)/Cu_2_O(111) heterojunction are shown in the top panels of Figure 13. We found that PF_5_ reduces its symmetry from the *D*_3*h*_ point group for the isolated molecule to *C*_4*v*_ upon adsorption, resulting in a change from a trigonal bipyramidal to a square pyramidal molecular geometry. The modification allows the central P atom to directly coordinate the exposed ridge 3-fold O anion at a distance of 1.75 Å and one of the F atoms to form a secondary interaction with a surface 3-fold Cu atom at 2.19 Å, thereby explaining the strong interaction observed for this adsorbate. Our calculations suggest that NO_2_, C_3_H_6_O_2_, and C_4_H_10_O_2_ molecules do not undergo significant distortion after adsorption, with respect to the configurations of their isolated states, as their point groups remained *C*_2*v*_, *C*_1_, and *C*_2*h*_, respectively. The NO_2_ and C_3_H_6_O_2_ adsorbates formed single interfacial coordinate N–O and Cu–O bonds at 1.47 and 2.12 Å, respectively, whereas C_4_H_10_O_2_ formed three weaker Cu–O interactions with an average distance of 2.69 Å. The molecular plane of C_3_H_6_O_2_, which aligns perpendicularly to the surface, and the carbon chain of C_4_H_10_O_2_ tend to orient themselves along the grooves in the [21̅1̅] direction. The H_2_O molecule, which retains its *C*_2*v*_ point group after adsorption, binds the 3-fold Cu cations perpendicularly to the surface grooves in a bent configuration and forms a hydrogen bond to the ridge 3-fold O anions. The analysis of the Bader charges indicates that only PF_5_ gained electron density from the binary heterostructure, whereas the other adsorbates became positively charged; see Table 1. The largest charge transfers were calculated for the adsorption sites that released the largest adsorption energies, i.e., the 3-fold Cu position for the organic molecules and H_2_O and the ridge 3-fold O position for PF_5_. The electron density of −0.142 e gained by PF_5_, which is the strongest adsorbed molecule, and 0.079 e donated by H_2_O, which is the weakest binding species, are in excellent accord with their adsorption energies. However, we found evidence of an inversely proportional relationship between the charge transfers and binding strengths for the most stable interaction configurations of the organic molecules and NO_2_ with the CuO(1̅11)/Cu_2_O(111) heterojunction. The bottom panels of Figure 13 illustrate the charge density difference (Δρ) for the adsorption modes that released the largest adsorption energies, where we also have indicated the atoms that underwent the most significant variation in their electron density. The surface O anions gained electron density, which was partially compensated by the Cu cations that lost electron charge upon interaction with C_3_H_6_O_2_, C_4_H_10_O_2_, and H_2_O, i.e., the adsorbates that are positively charged, and PF_5_. In contrast to the adsorptions where the molecules lost charge, our calculations imply that the interaction of NO_2_ provided 0.066 e to a nearby Cu atom, whereas the O adsorption site of the binary heterojunction donated 0.111 e. The differences in charge density indicate that the organic molecules received charge mainly from the H atoms, while a portion of the charge was redistributed to the C atoms. The central N and P atoms received significant charge donations of 0.223 and 0.194 e, respectively. Meanwhile, the terminal O atoms of NO_2_ and F atoms of PF_5_ gained electron density. Although the O atom of H_2_O experienced only minor charge gain, the H atoms lost 0.032 and 0.051 e upon adsorption.

Figure 13 illustrates the most favorable molecular adsorption modes that were calculated for the battery components or their degassing products on the ternary heterostructure. One of the methyl groups of C_4_H_10_O_2_ suffers dehydrogenation, enabling the unsaturated carbon and dissociated hydrogen atoms to directly coordinate surface O anions, thereby explaining the largest adsorption strength calculated in this study. The NO_2_, C_3_H_6_O_2_, PF_5_, and H_2_O adsorbates display similar adsorption structures in both the binary and ternary heterojunctions. However, C_3_H_6_O_2_, PF_5_, and H_2_O prefer to be located further away from the ternary than from the binary heterostructure, whereas the opposite was observed for NO_2_, which supports the relative adsorption energies of these adsorbates. Our calculations exhibit a good correlation between the trend of adsorption energies and Bader charge transfers calculated for the adsorbates upon interaction with TiO_2_(111)/CuO(1̅11)/Cu_2_O(111), as shown in Table 1. We found that only PF_5_ increased its electron density upon interaction with the ternary heterostructure, while the increasing order of the absolute value of charge transfers is
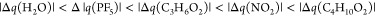
17

**Figure 14:**
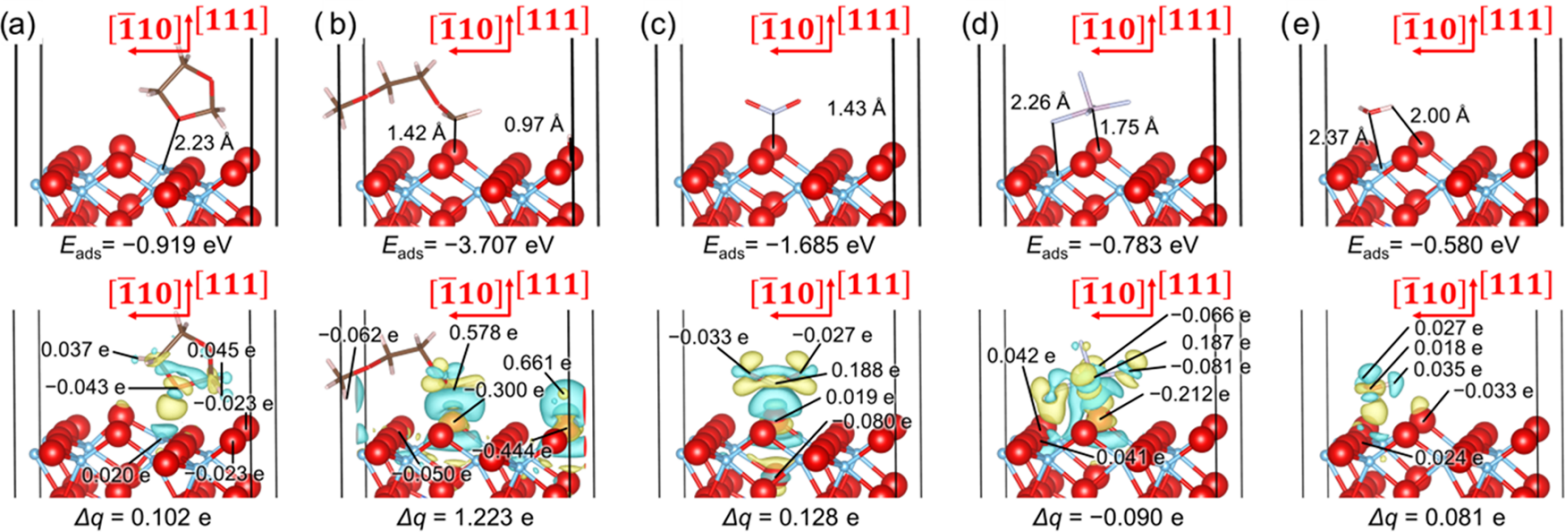
Adsorption of (a) C_3_H_6_O_2_, (b) C_4_H_10_O_2_, (c) NO_2_, (d) PF_5_, and (e) H_2_O on the TiO_2_(111)/CuO(1̅11)/Cu_2_O(111) heterojunction. Interatomic distances are shown in the top panels, while the charge density flow (Δρ) is represented in the bottom panels. Electron density gain and depletion regions are shown in yellow and green, respectively. Isosurfaces display a value of ±0.003 e Å^–3^. Charge transfers (Δ*q*) and atomic charge density differences for the atoms that suffered the largest change are also indicated. A negative value of charge transfer denotes that the adsorbate gains electron charge. Crystallographic directions are indicated with respect to the Cu_2_O(111) substrate. The ternary heterostructures are displayed using the ball-and-stick representation, whereas the adsorbates are shown using the stick representation. O atoms are shown in red, H atoms are shown in white, C atoms are shown in brown, F atoms are shown in light blue, P atoms are shown in pink, Cu atoms are shown in dark blue, and Ti atoms are shown in light blue.

The charge density difference for the most exothermic molecular interactions with TiO_2_(111)/CuO(1̅11)/Cu_2_O(111), alongside the atoms that experienced the largest change of electron charge, are displayed in the bottom panels of Figure 14. Similar to the binary heterojunction, the O anions of the ternary heterostructure showed an inclination to gain charge, whereas the Ti cations donated electron density upon interaction with the organic molecules. However, the adsorption of NO_2_ withdrew 0.019 e from the surface O site, donating 0.080 e to the O atom in the subsurface layer. As expected, the O adsorption site of PF_5_ gained 0.212 e, but the nearby O atoms lost 0.040 e, while the O atom forming the hydrogen bond with H_2_O received 0.033 e and a neighboring O anion donated 0.024 e. The interacting C from C_4_H_10_O_2_, N from NO_2_, and P from PF_5_ lost 0.578, 0.188, and 0.187 e, respectively, while the other atoms from those adsorbates gained smaller charge densities. Our charge density difference analysis indicates that the dissociated H from C_4_H_10_O_2_ also donated 0.661 e. The C from C_3_H_6_O_2_, which coordinated the interface, increased its electron density by 0.043 e, but the H atoms from the neighboring CH_2_ groups donated 0.037 and 0.045 e. We found that the three atoms from the H_2_O molecule donated minor charges to the ternary heterojunction.

**Figure 15:**
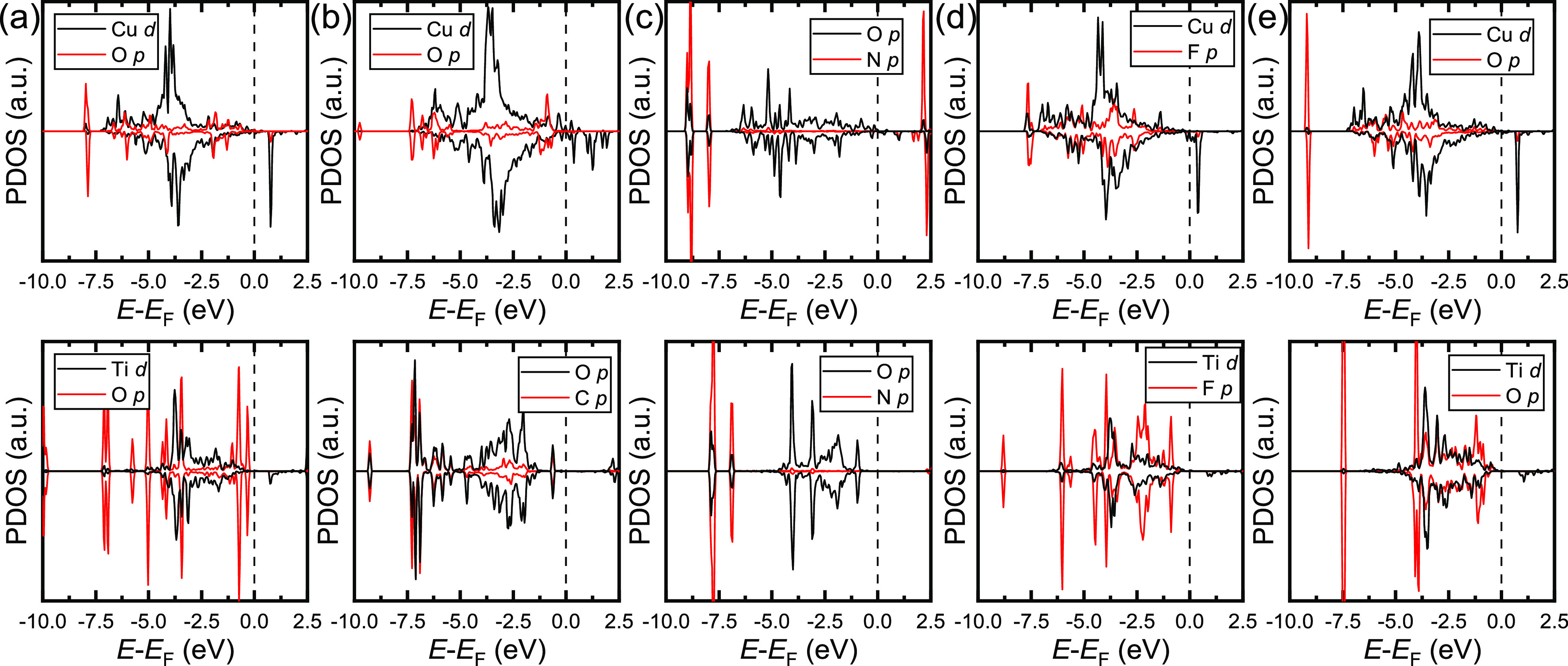
Atomic projections of the density of states (PDOS) for the most stable adsorption modes of (a) C_3_H_6_O_2_, (b) C_4_H_10_O_2_, (c) NO_2_, (d) PF_5_, and (e) H_2_O on the CuO(1̅11)/Cu_2_O(111) interface (top panels) and TiO_2_(111)/CuO(1̅11)/Cu_2_O(111) interface (bottom panels). The PDOS are only plotted for the interacting atoms, i.e., the d electrons of the Cu and Ti ions, the p states of the O, N, P, and F atom*s*, and the s orbitals of H atoms. The vertical dashed lines represent the Fermi level.

We have also calculated the projected density of states (PDOS) for the interacting atoms in the most thermodynamically stable adsorption modes. The top panels of Figure 15a,b,d,e indicate that the occupied d states of Cu have very similar characteristics in both spin channels for the interactions of C_3_H_6_O_2_, C_4_H_10_O_2_, PF_5_, and H_2_O, respectively, with the binary heterojunction. For example, the occupied d states of Cu are centered around −3.8 eV, with bands observed between −7.5 eV and the Fermi level. However, the unoccupied d levels of Cu in the minority spin channel only display a sharp peak at 1.0 eV for the interactions with C_3_H_6_O_2_, PF_5_, and H_2_O, making the interface a large energy gap insulator, whereas the band gap vanishes at the Fermi level for the adsorption of C_4_H_10_O_2_. Our calculations suggest that there is strong hybridization between the molecular O p orbitals and the Cu d states at around −6.3 and −2.0 eV in both spin channels, supporting the chemisorption modes calculated for C_3_H_6_O_2_, C_4_H_10_O_2_, and H_2_O on CuO(1̅11)/Cu_2_O(111). The interaction of PF_5_ with the binary interface leads to a broad hybridization of the F p states with the d levels of Cu, which extends over the entire valence band. The surface O anion coordinated by the NO_2_ molecule has an occupied band between −7.0 eV and the Fermi level in the majority channel of spins, as shown in the top panel of Figure 15c. Our calculations show that the minority spin channel splits into valence orbitals from −7.0 to −1.3 eV and conduction states around 1.1 eV. Interestingly, we also found a strong overlap in both channels of the spins between the highly localized orbitals at −8.8, −7.6, and 2.4 eV of the interacting nonmetallic O atom of the surface and N from NO_2_. The PDOS of the surface Ti d states and p orbitals of the O anions from the TiO_2_(111)/CuO(1̅11)/Cu_2_O(111) interface, alongside the interacting atoms from the adsorbates, are shown in the bottom panels of Figure 15. The Ti d band, which a center position calculated at approximately −3.8 eV, is less spread out than the Cu d bands for the interactions with C_3_H_6_O_2_, PF_5_, and H_2_O. The p states of the O atom of C_3_H_6_O_2_ display a series of discrete peaks in both channels of the spins of the valence band between −7.5 eV and the Fermi level, but only the peak observed at −3.7 eV overlaps with the Ti d band. Moreover, the F p orbital of PF_5_ and O p level of H_2_O are able to hybridize the entire Ti d states from −5.0 eV to the Fermi level. The p bands of the surface O atoms that interact with C_4_H_10_O_2_ are located between −7.5 eV and the valence band maximum (VBM) at −1.0 eV. Meanwhile, the main anion band contracts by 2.5 eV, with satellite peaks at −7.0 and −8.0 eV after adsorption of NO_2_. However, the bands located at −7.5 eV have the largest orbital overlap with the interacting atoms, C and N.

Our first-principles calculations of the interaction between vapors produced by battery solvents or their degassing products align with our gas response experiments. We have found that the binary heterojunction has a smaller preference than the ternary interface for the adsorbates. The binary CuO(1̅11)/Cu_2_O(111) sensor shows selectivity toward PF_5_, whereas C_4_H_10_O_2_ has the most favorable interaction with the ternary TiO_2_(111)/CuO(1̅11)/Cu_2_O(111) material. These interactions owe their large adsorption energies to the distortions experienced by the molecules upon interaction with the surfaces. For example, PF_5_ becomes a square pyramid, whereas one of the methyl groups of C_4_H_10_O_2_ suffers dehydrogenation. The simulated PDOS illustrates efficient hybridization between the bands of the interacting atoms from the interfaces and adsorbates. The Bader analysis shows that the O atom of the binary heterojunction donates charge to the electron-deficient P atom of PF_5_, while the C_4_H_9_O_2_ fragment transfers electron density to the O anion of the ternary sensor, explaining the formation of covalent bonds at the interfaces.

## 4 Conclusions

In this work, we have studied for the first time the use of CuO/Cu_2_O and TiO_2_/CuO/Cu_2_O heterostructures as 2-in-1 sensors for battery solvents and their degassing products. Scanning electron microscopy (SEM) has revealed that the heterostructures consist of well-packed layered nanocrystallites, which transform into nanogranules upon coating with a layer of TiO_2_ to form ternary heterostructures. EDX demonstrated the uniform distribution of the chemical elements. Using UV–vis spectroscopy, we found that these heterostructures possess direct optical band gaps, making them suitable for sensing applications owing to direct recombination with energy release. The sensing properties of CuO/Cu_2_O heterostructures with thicknesses of 10 nm (Cu10) showed higher sensitivity for the LP30 vapor with response values of ∼45% at operating temperatures of 250 and 300 °C. For TiO_2_/CuO/Cu_2_O heterostructures with different thicknesses, we observed a change in selectivity from LP30 vapors to C_4_H_10_O_2_ vapors. The optimal thickness of the Cu layer was found to be 10 nm, with response values of ∼70 and ∼90% at operating temperatures of 300 and 350 °C, respectively. We were also able to detect small concentrations (1 ppm) of C_4_H_10_O_2_ vapors, with response values of ∼7% at operating temperatures of 350 °C for three-layered structures with a thickness of 10 and 30 nm.

This work also reports the binding energy, interaction configurations, charge transfers, and PDOS for the adsorption of C_3_H_6_O_2_, C_4_H_10_O_2_, NO_2_, PF_5_, and H_2_O at the exposed surface of binary CuO(1̅11)/Cu_2_O(111) and ternary TiO_2_(111)/CuO(1̅11)/Cu_2_O(111) heterojunctions using first-principles methods. We have found that all molecular adsorptions are exothermic processes, suggesting that the sensors are able to detect all of the adsorbates. The ternary TiO_2_(111)/CuO(1̅11)/Cu_2_O(111) heteroepitaxial junction causes the largest adsorption energies to be released, which confirms the larger sensitivity found for this material in the gas response experiments. The interrogation of the thermodynamic preference of the molecules for the interfaces reveals that H_2_O will not replace the other adsorbates under competitive adsorption conditions, suggesting that humidity will not affect the performance of the sensors. The surface O atom was found to be the most reactive site, showing the largest selectivity toward PF_5_ and C_4_H_10_O_2_ in binary and ternary heterostructures, respectively. Despite the preference of the adsorbates, except for PF_5_, to donate electron density to the binary and ternary interfaces, our simulations of the PDOS demonstrated efficient overlap of the orbitals from the interacting atoms of the molecules and heterojunctions. The results acquired from this study can be used to tune the synthesis parameters of layered structured nanomaterials, such as doping or mixing content, to control the response to battery gas, battery solvents, or their degassing products, even at room-temperature operation. At the same time, the sensor works almost independently of the type of battery. This work and this sensor are also of interest in the use or development of solid-state batteries, since DOL is also a solvent typically used in solid-state batteries. These materials can be developed for use in new sensors and integrated devices for personal, industrial, safety and environmental applications.

## Supporting Information Available

EDX layered image and elemental compositional mapping images at the microstructural level of the CuO layer and TiO_2_/CuO heterostructures; EDX line scan profiles of O, Cu, and Ti taken along the TiO_2_/CuO heterostructures are also presented; and current–voltage characteristics of CuO/Cu_2_O and TiO_2_/CuO/Cu_2_O specimens with thicknesses of 50 nm (Cu50) measured at different temperatures (PDF)
