# Supplementary material for: Development of 2-in-1 Sensors for the Safety Assessment of Lithium-Ion Batteries via Early Detection of Vapors Produced by Electrolyte Solvents
Source: ACS Appl Mater Interfaces. 2023 May 26;15(22):27340–56. doi: 10.1021/acsami.3c03564 (PMC10251352; doi:10.1021/acsami.3c03564)
Supplement: Supplementary file 1 — am3c03564_si_001.pdf [file am3c03564_si_001.pdf]

Supporting Information

Development of 2-in-1 Sensors for the Safety Assessment of Lithium-Ion Batteries via Early Detection of Vapors Produced by Electrolyte Solvents

Oleg Lupan,[1,2,*] Nicolae Magariu,[2] David Santos-Carballal,[3,*] Nicolai Ababii,[2] Jakob Offermann,[1] Pia Pooker,[1] Sandra Hansen,[1,*] Leonard Siebert,[1,*] Nora H de Leeuw,[3,4] Rainer Adelung[1]

1 *Department for Materials Science – Functional Nanomaterials, Faculty of Engineering, Christian-Albrechts-University of Kiel, Kaiserstraße 2, D-24143 Kiel, Germany*
2 *Department of Microelectronics and Biomedical Engineering, Center for Nanotechnology and Nanosensors, Technical University of Moldova, 168 Stefan cel Mare Av., MD-2004 Chisinau, Republic of Moldova*
3 *School of Chemistry, University of Leeds, Leeds LS2 9JT, United Kingdom*
4 *Department of Earth Sciences, Utrecht University, Budapestlaan 4, 3584 CD Utrecht, The Netherlands*

* Corresponding authors:

Prof. Dr. O. Lupan, (ollu@tf.uni-kiel.de ; oleg.lupan@mib.utm.md)
Kiel University, Germany; Technical University of Moldova, Republic of Moldova

Dr. David Santos-Carballal (d.santos-carballal@leeds.ac.uk)
University of Leeds, United Kingdom.

Dr. Sandra Hansen, (sn@tf.uni-kiel.de)
Kiel University, Germany

Dr. Leonard Siebert, (lesi@tf.uni-kiel.de)
Kiel University, Germany

Text S1

The binary $CuO(\bar{1}11)/Cu_2O(111)$ and ternary $TiO_2(111)/CuO(\bar{1}11)/Cu_2O(111)$ heterostructures and their reactivity towards $C_3H_6O_2$, $C_4H_{10}O_2$, NO_2, PF_5 and H_2O were simulated using the Vienna Ab Initio Simulation Package (VASP) [1–4] We employed the simple generalized gradient approximation (GGA) exchange–correlation functional developed by Perdew, Burke and Ernzerhof (PBE) [5,6] for all our calculations, within the unrestricted formalism of DFT. We have chosen a kinetic energy cut-off of 400 eV for the periodic plane-wave basis set used to expand the Kohn–Sham (KS) valence states. The KS Hamiltonian was diagonalised using a combination of the blocked Davidson iteration scheme [7–9] for the initial phase and switched to the residual minimization method direct inversion in the iterative subspace (RMM-DIIS) [10] until the energy difference in two consecutive self-consistent loop steps dropped below 10^{-5} eV. The $3d4s$ levels of Cu and Ti, $3s3p$ levels of P, $2s2p$ levels of F, O, N and C, and $1s$ level of H were treated as valence states. The frozen core electrons and their interaction with the valence levels were modelled using the projected augmented wave (PAW) formalism [11,12], which also includes the non-spherical contributions of the density gradient within the one-centre terms. An efficient Pulay method was used for mixing the charge densities [10], including up to the *g* orbitals of the one-centre PAW charge densities, whereas the charge-dielectric function proposed by Kerker was employed for the initial approximation [13]. A consistent and accurately parametrised long-range dispersion correction with a Becke and Johnson damping function [D3-(BJ)] [14,15] was added to the standard Kohn-Sham DFT energy, to obtain better non-bonded distances and non-covalent interaction energies [16–24]. A constrained GGA Hubbard parameter [25,26] U_{eff} = 4.0 eV for Cu and 4.4 eV for Ti was used to enhance the description of the electron correlations in the $3d$ shell of the cations in the bulk phases of Cu_2O, CuO and TiO_2 [27,28]. The geometry optimisations were carried out using a Newton line optimiser, which is an efficient conjugate gradients technique [29,30], with a maximum allowed step size of λ = 0.2 Å and a finite difference step size of 5×10^{-3} Å applied to calculate the curvature. The geometry optimisations stopped when the

Hellmann-Feynman forces on all atoms reached the minimum threshold of 0.01 eV $Å^{-1}$. These settings resulted in the convergence of the total electronic energy to within 1 meV $atom^{-1}$.

The electronic integrations of the interfaces both before and after molecular adsorptions were calculated in the reciprocal space using Γ-centered Monkhorst−Pack (MP) grids [31] of $5 \times 5 \times 1$ k-points, in agreement with earlier studies [27,28,32]. The simulations of the isolated adsorbate molecules were performed sampling only the Γ point of the Brillouin zone (BZ), using a cell with dimensions of $20 \times 21 \times 22$ $Å^3$ and broken symmetry. The electronic partial occupancies during geometry optimisations for all our systems were determined using the Gaussian smearing [33–36], with a sigma value σ = 0.05 eV. However, the tetrahedron method with Blöchl corrections [37–41] was used in final static calculations to obtain accurate energies and electronic properties for the optimised structure of systems that were described by more than a single k-point. Dipole corrections were applied in the direction perpendicular to the surface plane [42,43] to account for any dipole created by the interaction with the adsorbates on the relaxed side of the slab and to improve the electronic convergence [16,19,28,44,45].

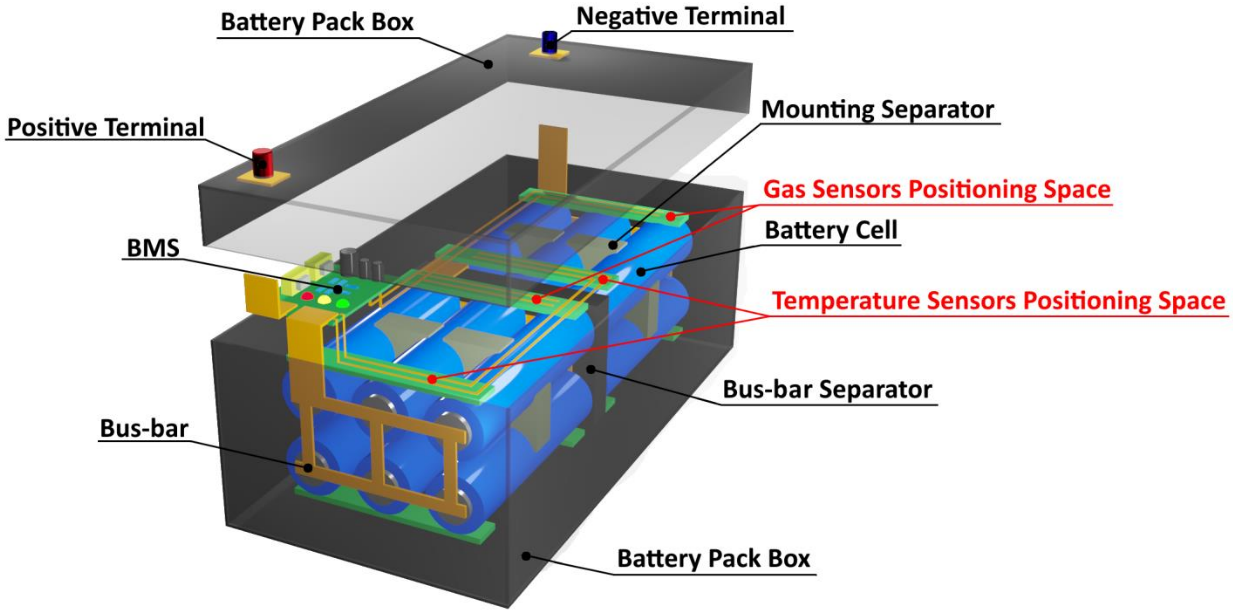

Figure S1. Schematic concept of the battery pack

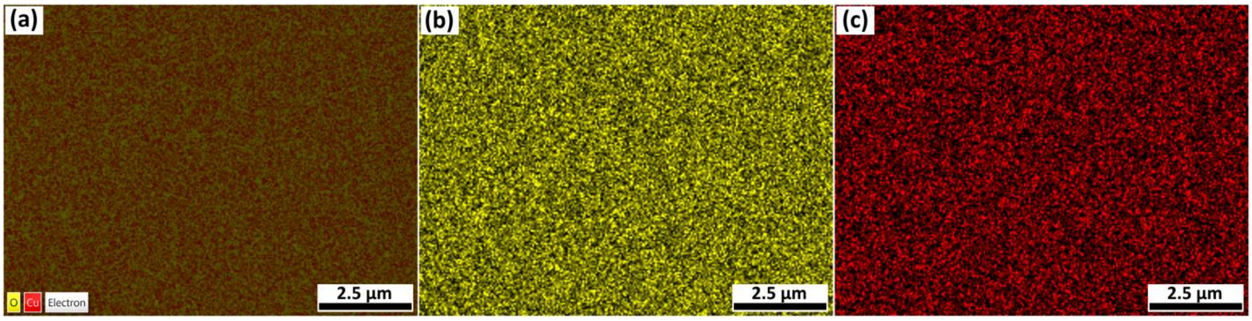

Figure S2. (a) Layered image and compositional images obtained through EDX elemental mapping at the microstructural level of the CuO layer for (b) O Kα1; and (c) Cu Lα1_2 distributions.

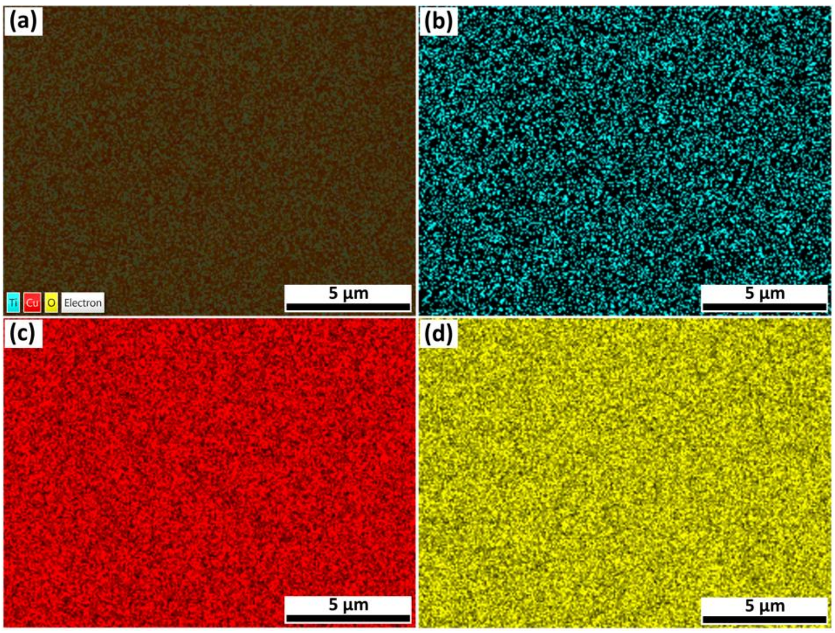

Figure S3. (a) Layered image and compositional images obtained through EDX elemental mapping at the microstructural level of the TiO_2/CuO heterostructures for (b) Ti Kα1; (c) Cu Lα1_2; and (d) O Kα1 distributions.

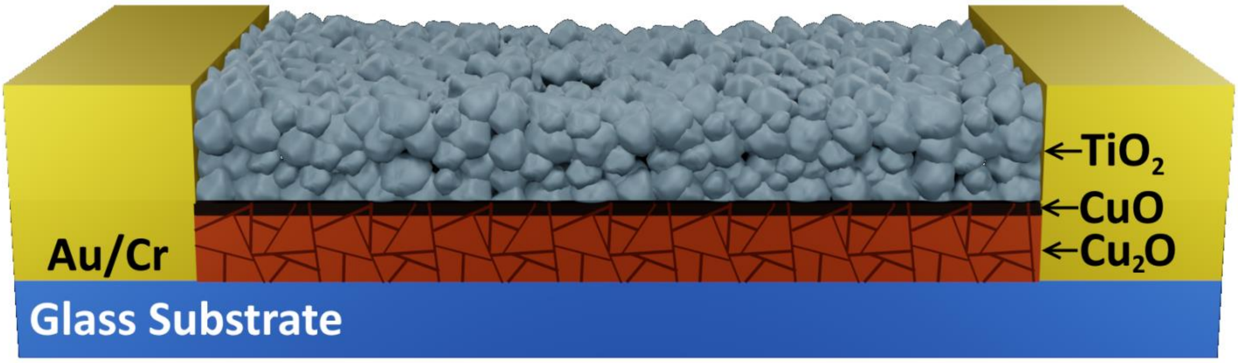

Figure S4. Schematic sectional-view of ternary $TiO_2(111)/CuO(\bar{1}11)/Cu_2O(111)$ heterojunction.

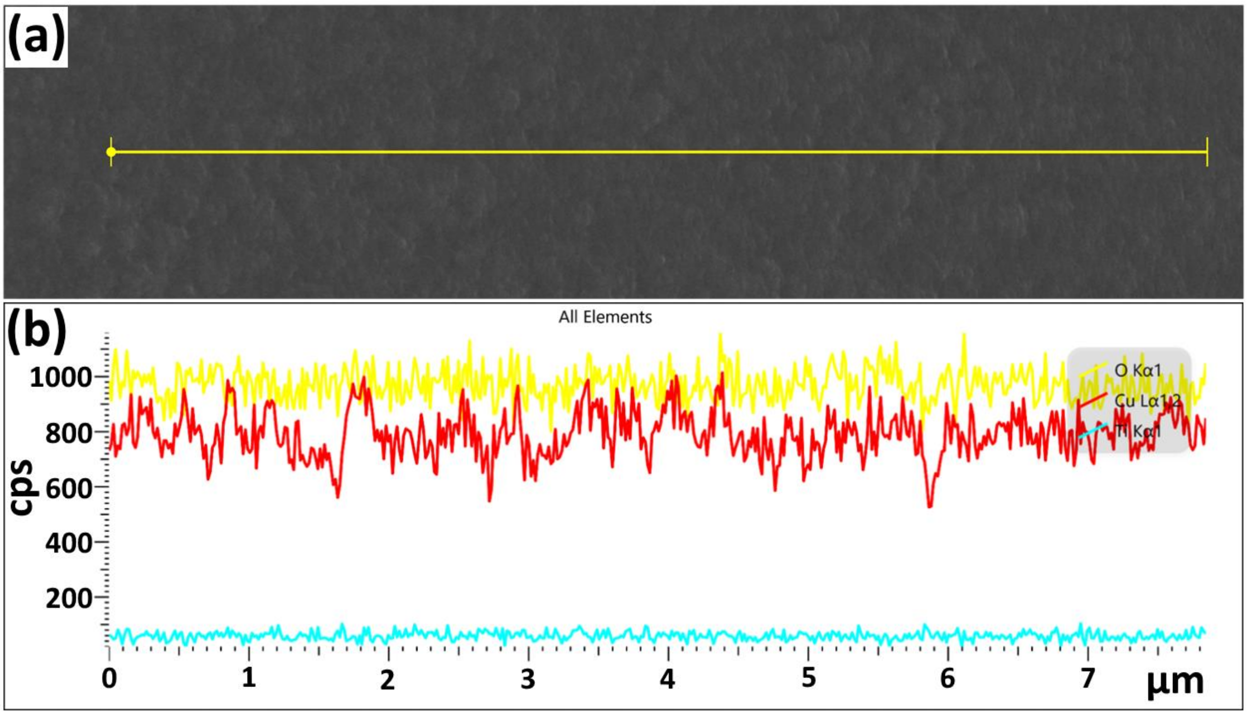

Figure S5. (a) SEM image and (b) EDX-line scan profiles of O, Cu and Ti taken along the TiO_2/CuO heterostructures shown in (a).

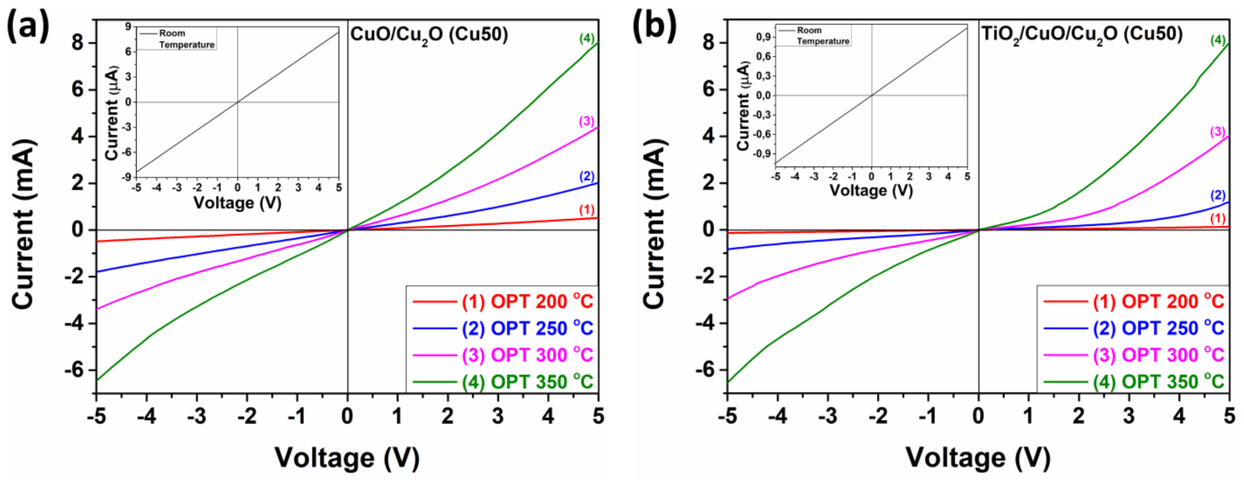

Figure S6. Current-voltage (I-V) characteristics of (a) CuO/Cu_2O samples and (b) TiO_2/CuO/Cu_2O samples with a thickness of 50 nm (Cu50), measured at different temperatures.

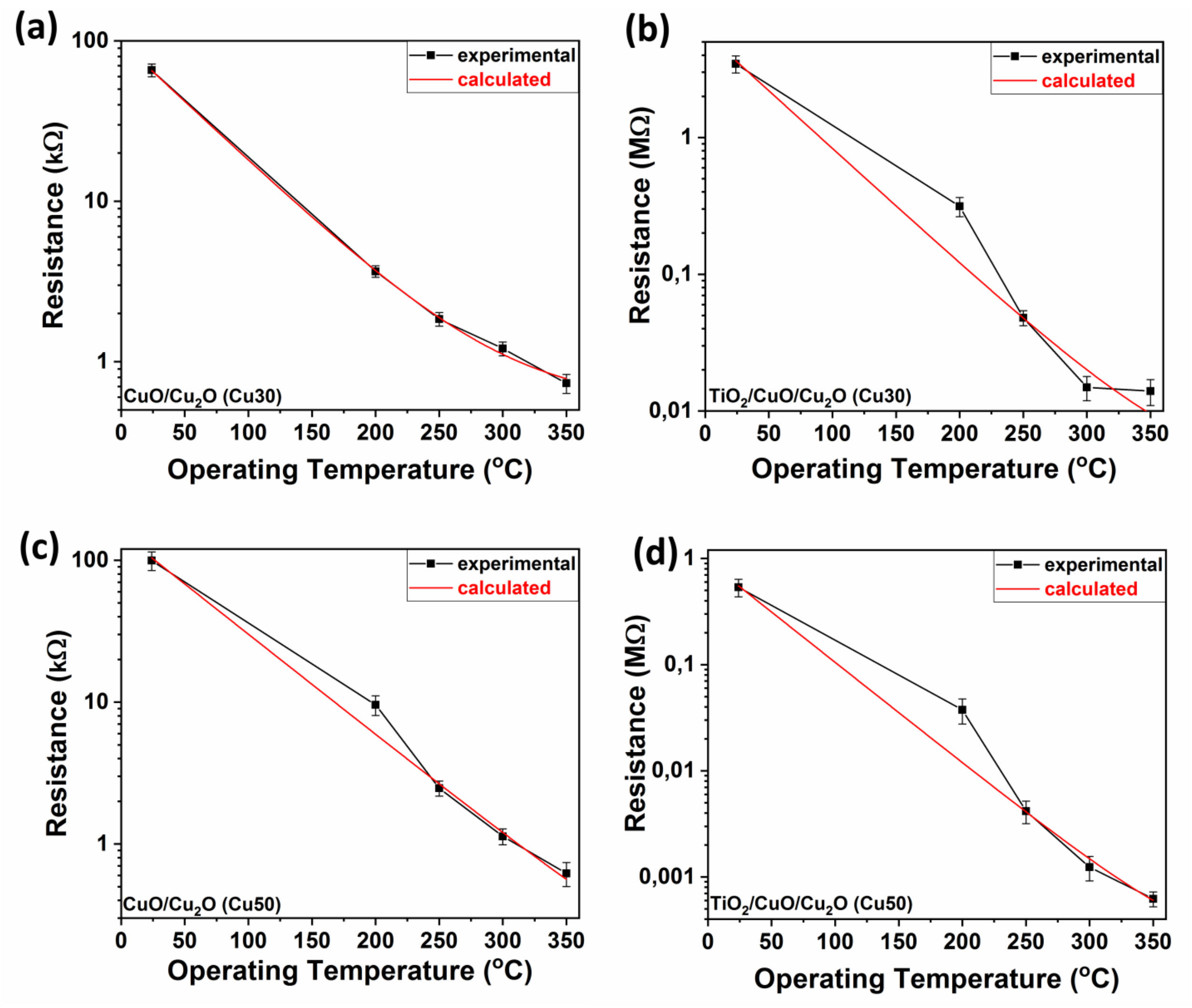

Figure S7. Dependence of electrical resistance versus operating temperature for (a) CuO/Cu_2O (Cu30); (b) $TiO_2/CuO/Cu_2O$ (Cu30); (c) CuO/Cu_2O (Cu50); and (d) $TiO_2/CuO/Cu_2O$ (Cu50).

Table S1. Gas sensors suitable for detecting gases released by batteries

Sensing structure	Target gas	Gas response	Concentration (ppm)	Operating temperature (ºC)	Year of publication
Al-ZnO	CO	1.8	50	300	2014[46]
GaN	H_2	101.5	750	500	2018[47]
ZIF-67-SnO_2	CO_2	16.5	5000	205	2018[48]
Pd-SnO_2	C_2H_4	28.69	100	350	2019[49]
CeO_x-SnO_2	C_2H_4	5.18	10	350	2020[50]
Au-ZnO	CH_4	4.16	100	250	2020[51]
IC-MOFs	DMC	55%	200	-	2020[52]
Graphene	NO_2	2.95%	100	-	2020[53]
Al_2O_3/CuO-3D	$C_3H_6O_2$	10%	100	200	2021[54]
	$C_4H_{10}O_2$	16%	100	300	
CuO:Fe_2O_3-3D	$C_3H_6O_2$	28%	100	250	
	$LiNO_3$	46%	100	350	
PV3D3/CuO	$C_4H_{10}O_2$	33%	100	350	2022[55]
**TiO_2/CuO/Cu_2O**	**$C_4H_{10}O_2$**	**89%**	**100**	**350**	**This work**
	**$LiPF_6$**	**46%**	**100**	**250**	

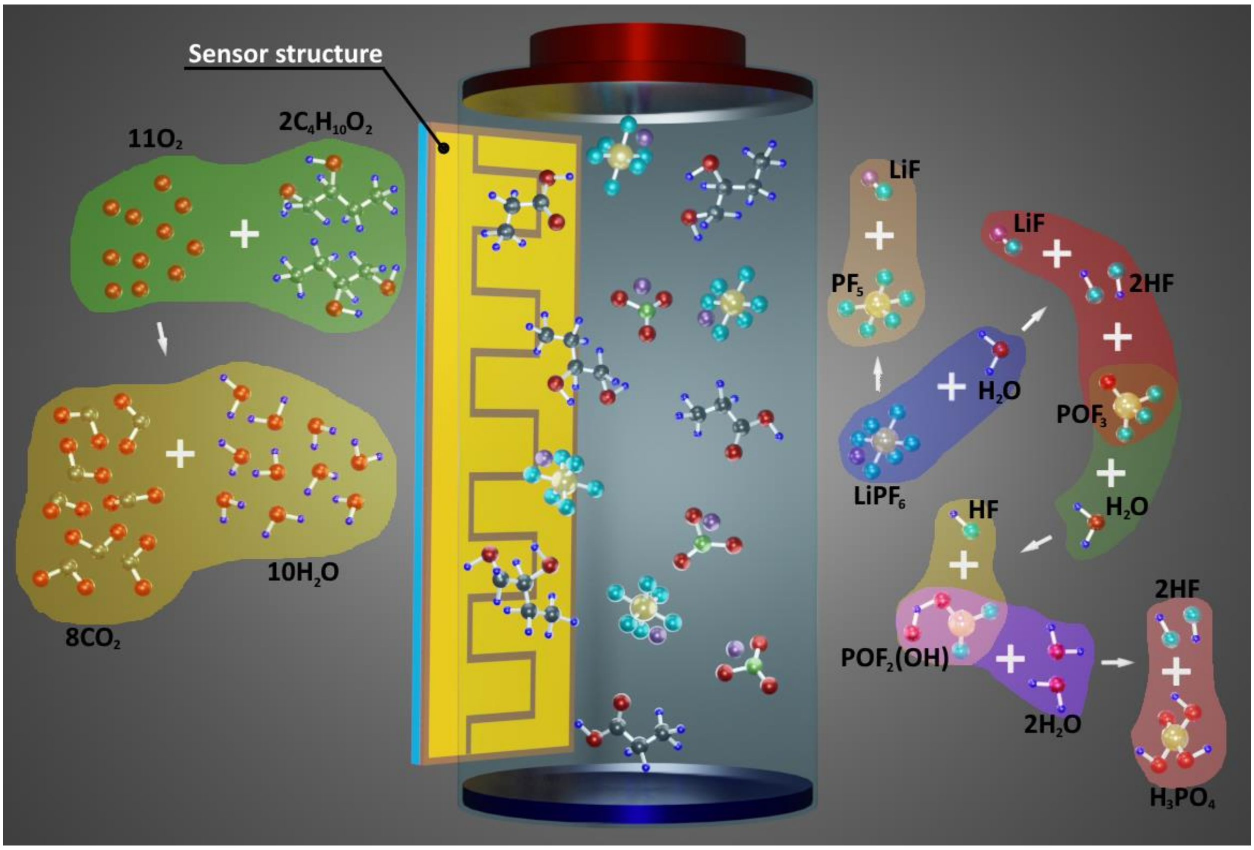

Figure S8. Schematic representation of the interaction processes between $LiPF_6$ and $C_4H_{10}O_2$ with the environmental species.

References

(1) Kresse, G.; Hafner, J. Ab Initio Molecular Dynamics for Liquid Metals. *Phys. Rev. B* **1993**, *47* (1), 558–561.

(2) Kresse, G.; Hafner, J. Ab Initio Molecular-Dynamics Simulation of the Liquid-Metal–Amorphous-Semiconductor Transition in Germanium. *Phys. Rev. B* **1994**, *49* (20), 14251–14269.

(3) Kresse, G.; Furthmüller, J. Efficiency of Ab-Initio Total Energy Calculations for Metals and Semiconductors Using a Plane-Wave Basis Set. *Comput. Mater. Sci.* **1996**, *6* (1), 15–50.

(4) Kresse, G.; Furthmüller, J. Efficient Iterative Schemes for Ab Initio Total-Energy Calculations Using a Plane-Wave Basis Set. *Phys. Rev. B* **1996**, *54* (16), 11169–11186.

(5) Perdew, J. P.; Burke, K.; Ernzerhof, M. Generalized Gradient Approximation Made Simple. *Phys. Rev. Lett.* **1997**, *78* (7), 1396–1396.

(6) Perdew, J. P.; Burke, K.; Ernzerhof, M. Generalized Gradient Approximation Made Simple. *Phys. Rev. Lett.* **1996**, *77* (18), 3865–3868.

(7) Davidson, E. R. Matrix Eigenvector Methods. In *Methods in Computational Molecular Physics*; Diercksen, G. H. F., Wilson, S., Eds.; Springer Netherlands: Dordrecht, 1983; pp 95–113.

(8) Davidson, E. R. The Iterative Calculation of a Few of the Lowest Eigenvalues and Corresponding Eigenvectors of Large Real-Symmetric Matrices. *J. Comput. Phys.* **1975**, *17* (1), 87–94.

(9) Liu, B. The Simultaneous Expansion-Method For The Iterative Solution Of Several Of The Lowest Eigenvalues And Corresponding Eigenvectors Of Large Real-Symmetric Matrices. In *National Resource for Computation in Chemistry*; Moler, C., Shavitt, I., Eds.; California, Berkeley, 1978; pp 49–53.

(10) Pulay, P. Convergence Acceleration of Iterative Sequences. the Case of Scf Iteration. *Chem. Phys. Lett.* **1980**, *73* (2), 393–398.

(11) Blöchl, P. E. Projector Augmented-Wave Method. *Phys. Rev. B* **1994**, *50* (24), 17953–17979.

(12) Kresse, G.; Joubert, D. From Ultrasoft Pseudopotentials to the Projector Augmented-Wave Method. *Phys. Rev. B* **1999**, *59* (3), 1758–1775.

(13) Kerker, G. P. Efficient Iteration Scheme for Self-Consistent Pseudopotential Calculations. *Phys. Rev. B* **1981**, *23* (6), 3082–3084.

(14) Grimme, S.; Antony, J.; Ehrlich, S.; Krieg, H. A Consistent and Accurate Ab Initio Parametrization of Density Functional Dispersion Correction (DFT-D) for the 94 Elements H-Pu. *J. Chem. Phys.* **2010**, *132* (15), 154104.

(15) Grimme, S.; Ehrlich, S.; Goerigk, L. Effect of the Damping Function in Dispersion Corrected Density Functional Theory. *J. Comput. Chem.* **2011**, *32* (7), 1456–1465.

(16) Santos-Carballal, D.; Cadi-Essadek, A.; de Leeuw, N. H. Catalytic Conversion of CO and H_2 into Hydrocarbons on the Cobalt Co(111) Surface: Implications for the Fischer–Tropsch Process. *J. Phys. Chem. C* **2021**, *125* (22), 11891–11903.

(17) Cadi-Essadek, A.; Roldan, A.; Santos-Carballal, D.; Ngoepe, P. E.; Claeys, M.; de Leeuw, N. H. DFT+U Study of the Electronic, Magnetic and Mechanical Properties of Co, CoO, and Co_3O_4. *South African J. Chem.* **2021**, *74 (Specia*, 8–16.

(18) Botha, L. M.; Santos-Carballal, D.; Terranova, U.; Quesne, M. G.; Ungerer, M. J.; van Sittert, C. G. C. E.; de Leeuw, N. H. Mixing Thermodynamics and Electronic Structure of the $Pt_{1-x}Ni_x$ ($0 \leq x \leq 1$) Bimetallic Alloy. *RSC Adv.* **2019**, *9* (30), 16948–16954.

(19) Ramogayana, B.; Santos-Carballal, D.; Aparicio, P. A.; Quesne, M. G.; Maenetja, K. P.; Ngoepe, P. E.; de Leeuw, N. H. Ethylene Carbonate Adsorption on the Major Surfaces of Lithium Manganese Oxide $Li_{1-x}Mn_2O_4$ Spinel ($0.000 < x < 0.375$): A DFT+ U -D3 Study. *Phys. Chem. Chem. Phys.* **2020**, *22* (12), 6763–6771.

(20) Vahl, A.; Lupan, O.; Santos-Carballal, D.; Postica, V.; Hansen, S.; Cavers, H.; Wolff, N.;

Terasa, M.-I.; Hoppe, M.; Cadi-Essadek, A.; Dankwort, T.; Kienle, L.; de Leeuw, N. H.; Adelung, R.; Faupel, F. Surface Functionalization of ZnO:Ag Columnar Thin Films with AgAu and AgPt Bimetallic Alloy Nanoparticles as an Efficient Pathway for Highly Sensitive Gas Discrimination and Early Hazard Detection in Batteries. *J. Mater. Chem. A* **2020**, *8* (32), 16246–16264.
(21) Reguera, L.; López, N. L.; Rodríguez-Hernández, J.; González, M.; Hernandez-Tamargo, C. E.; Santos-Carballal, D.; de Leeuw, N. H.; Reguera, E. Synthesis, Crystal Structures, and Properties of Zeolite-Like $T_3(H_3O)_2M(CN)_6]_2\cdot uH_2O$ (T = Co, Zn; M = Ru, Os). *Eur. J. Inorg. Chem.* **2017**, *2017* (23), 2980–2989.
(22) Malatji, K. T.; Santos-Carballal, D.; Terranova, U.; Ngoepe, P. E.; de Leeuw, N. H. Controlling the Lithium Intercalation Voltage in the $Li(Mn_{1-x}Ni_x)_2O_4$ Spinel via Tuning of the Ni Concentration: A Density Functional Theory Study. *South African J. Chem.* **2021**, *74*, 3–7.
(23) Santos-Carballal, D.; Ngoepe, P. E.; de Leeuw, N. H. Ab Initio Investigation of the Thermodynamics of Cation Distribution and of the Electronic and Magnetic Structures in the $LiMn_2O_4$ Spinel. *Phys. Rev. B* **2018**, *97* (8), 085126.
(24) Posada-Pérez, S.; Santos-Carballal, D.; Terranova, U.; Roldan, A.; Illas, F.; de Leeuw, N. H. CO_2 Interaction with Violarite ($FeNi_2S_4$) Surfaces: A Dispersion-Corrected DFT Study. *Phys. Chem. Chem. Phys.* **2018**, *20* (31), 20439–20446.
(25) Dudarev, S. L.; Botton, G. A.; Savrasov, S. Y.; Humphreys, C. J.; Sutton, A. P. Electron-Energy-Loss Spectra and the Structural Stability of Nickel Oxide: An LSDA+U Study. *Phys. Rev. B* **1998**, *57* (3), 1505–1509.
(26) Anisimov, V. I.; Zaanen, J.; Andersen, O. K. Band Theory and Mott Insulators: Hubbard U Instead of Stoner I. *Phys. Rev. B* **1991**, *44* (3), 943–954.
(27) Lupan, O.; Ababii, N.; Santos-Carballal, D.; Terasa, M.-I.; Magariu, N.; Zappa, D.; Comini, E.; Pauporté, T.; Siebert, L.; Faupel, F.; Vahl, A.; Hansen, S.; de Leeuw, N. H.; Adelung, R. Tailoring the Selectivity of Ultralow-Power Heterojunction Gas Sensors by Noble Metal Nanoparticle Functionalization. *Nano Energy* **2021**, *88*, 106241.
(28) Lupan, O.; Santos-Carballal, D.; Ababii, N.; Magariu, N.; Hansen, S.; Vahl, A.; Zimoch, L.; Hoppe, M.; Pauporté, T.; Galstyan, V.; Sontea, V.; Chow, L.; Faupel, F.; Adelung, R.; de Leeuw, N. H.; Comini, E. $TiO_2/Cu_2O/CuO$ Multi-Nanolayers as Sensors for H_2 and Volatile Organic Compounds: An Experimental and Theoretical Investigation. *ACS Appl. Mater. Interfaces* **2021**, *13* (27), 32363–32380.
(29) Hestenes, M. R.; Stiefel, E. Methods of Conjugate Gradients for Solving Linear Systems. *Journal of Research of the National Bureau of Standards*. 1952, p 409.
(30) Sheppard, D.; Terrell, R.; Henkelman, G. Optimization Methods for Finding Minimum Energy Paths. *J. Chem. Phys.* **2008**, *128* (13), 134106.
(31) Monkhorst, H. J.; Pack, J. D. Special Points for Brillouin-Zone Integrations. *Phys. Rev. B* **1976**, *13* (12), 5188–5192.
(32) Cretu, V.; Postica, V.; Mishra, A. K.; Hoppe, M.; Tiginyanu, I.; Mishra, Y. K.; Chow, L.; de Leeuw, N. H.; Adelung, R.; Lupan, O. Synthesis, Characterization and DFT Studies of Zinc-Doped Copper Oxide Nanocrystals for Gas Sensing Applications. *J. Mater. Chem. A* **2016**, *4* (17), 6527–6539.
(33) Ho, K. M.; Fu, C. L.; Harmon, B. N.; Weber, W.; Hamann, D. R. Vibrational Frequencies and Structural Properties of Transition Metals via Total-Energy Calculations. *Phys. Rev. Lett.* **1982**, *49* (9), 673–676.
(34) Fu, C. L.; Ho, K. M. First-Principles Calculation of the Equilibrium Ground-State Properties of Transition Metals: Applications to Nb and Mo. *Phys. Rev. B* **1983**, *28* (10), 5480–5486.
(35) De Vita, A. The energetics of defects and impurities in metals and ionic materials from first principles. PhD Thesis, Keele University, 1992. https://ethos.bl.uk/OrderDetails.do?uin=uk.bl.ethos.332301 (accessed 2020-12-15).

(36) Needs, R. J.; Martin, R. M.; Nielsen, O. H. Total-Energy Calculations of the Structural Properties of the Group-V Element Arsenic. *Phys. Rev. B* **1986**, *33* (6), 3778–3784.
(37) Methfessel, M. S.; Boon, M. H.; Mueller, F. M. Analytic-Quadratic Method of Calculating the Density of States. *J. Phys. C Solid State Phys.* **1983**, *16* (27), L949–L954.
(38) Lehmann, G.; Taut, M. On the Numerical Calculation of the Density of States and Related Properties. *Phys. Status Solidi* **1972**, *54* (2), 469–477.
(39) Jepson, O.; Anderson, O. K. The Electronic Structure of h.c.p. Ytterbium. *Solid State Commun.* **1971**, *9* (20), 1763–1767.
(40) Gilat, G. Analysis of Methods for Calculating Spectral Properties in Solids. *J. Comput. Phys.* **1972**, *10* (3), 432–465.
(41) Blöchl, P. E.; Jepsen, O.; Andersen, O. K. Improved Tetrahedron Method for Brillouin-Zone Integrations. *Phys. Rev. B* **1994**, *49* (23), 16223–16233.
(42) Neugebauer, J.; Scheffler, M. Adsorbate-Substrate and Adsorbate-Adsorbate Interactions of Na and K Adlayers on Al(111). *Phys. Rev. B* **1992**, *46* (24), 16067–16080.
(43) Makov, G.; Payne, M. C. Periodic Boundary Conditions in Ab Initio Calculations. *Phys. Rev. B* **1995**, *51* (7), 4014–4022.
(44) Peck, M. A.; Santos-Carballal, D.; de Leeuw, N. H.; Claeys, M. Density Functional Theory Study of the Adsorption of Oxygen and Hydrogen on 3d Transition Metal Surfaces with Varying Magnetic Ordering. *South African J. Chem.* **2021**, *74 (Specia*, 69–72.
(45) Santos-Carballal, D.; Roldan, A.; Grau-Crespo, R.; de Leeuw, N. H. A DFT Study of the Structures, Stabilities and Redox Behaviour of the Major Surfaces of Magnetite Fe_3O_4. *Phys. Chem. Chem. Phys.* **2014**, *16* (39), 21082–21097.
(46) Hjiri, M.; El Mir, L.; Leonardi, S. G.; Pistone, A.; Mavilia, L.; Neri, G. Al-Doped ZnO for Highly Sensitive CO Gas Sensors. *Sensors Actuators B Chem.* **2014**, *196*, 413–420.
(47) Hermawan, A.; Asakura, Y.; Kobayashi, M.; Kakihana, M.; Yin, S. High Temperature Hydrogen Gas Sensing Property of GaN Prepared from α-GaOOH. *Sensors Actuators B Chem.* **2018**, *276*, 388–396.
(48) DMello, M. E.; Sundaram, N. G.; Kalidindi, S. B. Assembly of ZIF-67 Metal-Organic Framework over Tin Oxide Nanoparticles for Synergistic Chemiresistive CO_2 Gas Sensing. *Chem. - A Eur. J.* **2018**, *24* (37), 9220–9223.
(49) A. M. Akhir, M.; Rezan, S. A.; Mohamed, K.; Arafat, M. M.; Haseeb, A. S. M. A.; Lee, H. L. Synthesis of SnO_2 Nanoparticles via Hydrothermal Method and Their Gas Sensing Applications for Ethylene Detection. *Mater. Today Proc.* **2019**, *17*, 810–819.
(50) Leangtanom, P.; Wisitsoraat, A.; Jaruwongrungsee, K.; Chanlek, N.; Phanichphant, S.; Kruefu, V. Highly Sensitive and Selective Ethylene Gas Sensors Based on CeO_x-SnO_2 Nanocomposites Prepared by a Co-Precipitation Method. *Mater. Chem. Phys.* **2020**, *254*, 123540.
(51) Zhang, B.; Wang, Y.; Meng, X.; Zhang, Z.; Mu, S. High Response Methane Sensor Based on Au-Modified Hierarchical Porous Nanosheets-Assembled ZnO Microspheres. *Mater. Chem. Phys.* **2020**, *250*, 123027.
(52) Lu, Y.; Zhang, S.; Dai, S.; Liu, D.; Wang, X.; Tang, W.; Guo, X.; Duan, J.; Luo, W.; Yang, B.; Zou, J.; Huang, Y.; Katz, H. E.; Huang, J. Ultrasensitive Detection of Electrolyte Leakage from Lithium-Ion Batteries by Ionically Conductive Metal-Organic Frameworks. *Matter* **2020**, *3* (3), 904–919.
(53) Wang, H.; Wang, H.; Wang, Y.; Su, X.; Wang, C.; Zhang, M.; Jian, M.; Xia, K.; Liang, X.; Lu, H.; Li, S.; Zhang, Y. Laser Writing of Janus Graphene/Kevlar Textile for Intelligent Protective Clothing. *ACS Nano* **2020**, *14* (3), 3219–3226.
(54) Lupan, O.; Krüger, H.; Siebert, L.; Ababii, N.; Kohlmann, N.; Buzdugan, A.; Bodduluri, M. T.; Magariu, N.; Terasa, M.-I.; Strunskus, T.; Kienle, L.; Adelung, R.; Hansen, S. Additive Manufacturing as a Means of Gas Sensor Development for Battery Health Monitoring. *Chemosensors* **2021**, *9* (9), 252.

(55) Schröder, S.; Ababii, N.; Lupan, O.; Drewes, J.; Magariu, N.; Krüger, H.; Strunskus, T.; Adelung, R.; Hansen, S.; Faupel, F. Sensing Performance of $CuO/Cu_2O/ZnO$:Fe Heterostructure Coated with Thermally Stable Ultrathin Hydrophobic PV3D3 Polymer Layer for Battery Application. *Mater. Today Chem.* **2022**, *23*, 100642.
